# Dynamic changes in immune gene co-expression networks predict development of type 1 diabetes

**DOI:** 10.1038/s41598-021-01840-z

**Published:** 2021-11-22

**Authors:** Ingrid Brænne, Suna Onengut-Gumuscu, Ruoxi Chen, Ani W. Manichaikul, Stephen S. Rich, Wei-Min Chen, Charles R. Farber, the TEDDY Study Group, Marian Rewers, Aaron Barbour, Kimberly Bautista, Judith Baxter, Daniel Felipe-Morales, Kimberly Driscoll, Brigitte I. Frohnert, Marisa Stahl, Patricia Gesualdo, Michelle Hoffman, Rachel Karban, Edwin Liu, Jill Norris, Stesha Peacock, Hanan Shorrosh, Andrea Steck, Megan Stern, Erica Villegas, Kathleen Waugh, Jorma Toppari, Olli G. Simell, Annika Adamsson, Suvi Ahonen, Mari Åkerlund, Leena Hakola, Anne Hekkala, Henna Holappa, Heikki Hyöty, Anni Ikonen, Jorma Ilonen, Sinikka Jäminki, Sanna Jokipuu, Leena Karlsson, Jukka Kero, Miia Kähönen, Mikael Knip, Minna-Liisa Koivikko, Merja Koskinen, Mirva Koreasalo, Kalle Kurppa, Jarita Kytölä, Tiina Latva-aho, Katri Lindfors, Maria Lönnrot, Elina Mäntymäki, Markus Mattila, Maija Miettinen, Katja Multasuo, Teija Mykkänen, Tiina Niininen, Sari Niinistö, Mia Nyblom, Sami Oikarinen, Paula Ollikainen, Zhian Othmani, Sirpa Pohjola, Petra Rajala, Jenna Rautanen, Anne Riikonen, Eija Riski, Miia Pekkola, Minna Romo, Satu Ruohonen, Satu Simell, Maija Sjöberg, Aino Stenius, Päivi Tossavainen, Mari Vähä-Mäkilä, Sini Vainionpää, Eeva Varjonen, Riitta Veijola, Irene Viinikangas, Suvi M. Virtanen, Jin-Xiong She, Desmond Schatz, Diane Hopkins, Leigh Steed, Jennifer Bryant, Katherine Silvis, Michael Haller, Melissa Gardiner, Richard McIndoe, Ashok Sharma, Stephen W. Anderson, Laura Jacobsen, John Marks, P. D. Towe, Anette G. Ziegler, Ezio Bonifacio, Anita Gavrisan, Cigdem Gezginci, Anja Heublein, Verena Hoffmann, Sandra Hummel, Andrea Keimer, Annette Knopff, Charlotte Koch, Sibylle Koletzko, Claudia Ramminger, Roswith Roth, Marlon Scholz, Joanna Stock, Katharina Warncke, Lorena Wendel, Christiane Winkler, Åke Lernmark, Daniel Agardh, Carin Andrén Aronsson, Maria Ask, Rasmus Bennet, Corrado Cilio, Helene Engqvist, Emelie Ericson-Hallström, Annika Fors, Lina Fransson, Thomas Gard, Monika Hansen, Hanna Jisser, Fredrik Johansen, Berglind Jonsdottir, Silvija Jovic, Helena Elding Larsson, Marielle Lindström, Markus Lundgren, Marlena Maziarz, Maria Månsson-Martinez, Maria Markan, Jessica Melin, Zeliha Mestan, Caroline Nilsson, Karin Ottosson, Kobra Rahmati, Anita Ramelius, Falastin Salami, Anette Sjöberg, Birgitta Sjöberg, Malin Svensson, Carina Törn, Anne Wallin, Åsa Wimar, Sofie Åberg, William A. Hagopian, Michael Killian, Claire Cowen Crouch, Jennifer Skidmore, Rachel Hervey, Rachel Lyons, Arlene Meyer, Denise Mulenga, Matei Romancik, Davey Schmitt, Dorothy Becker, Margaret Franciscus, MaryEllen Dalmagro-Elias Smith, Ashi Daftary, Mary Beth Klein, Chrystal Yates, Jeffrey P. Krischer, Sarah Austin-Gonzalez, Maryouri Avendano, Sandra Baethke, Rasheedah Brown, Brant Burkhardt, Martha Butterworth, Joanna Clasen, David Cuthbertson, Stephen Dankyi, Christopher Eberhard, Steven Fiske, Jennifer Garmeson, Veena Gowda, Kathleen Heyman, Belinda Hsiao, Christina Karges, Francisco Perez Laras, Hye-Seung Lee, Qian Li, Shu Liu, Xiang Liu, Kristian Lynch, Colleen Maguire, Jamie Malloy, Cristina McCarthy, Aubrie Merrell, Hemang Parikh, Ryan Quigley, Cassandra Remedios, Chris Shaffer, Laura Smith, Susan Smith, Noah Sulman, Roy Tamura, Dena Tewey, Michael Toth, Ulla Uusitalo, Kendra Vehik, Ponni Vijayakandipan, Keith Wood, Jimin Yang, Michael Abbondondolo, Lori Ballard, David Hadley, Wendy McLeod, Steven Meulemans, Liping Yu, Dongmei Miao, Polly Bingley, Alistair Williams, Kyla Chandler, Olivia Ball, Ilana Kelland, Sian Grace, Masumeh Chavoshi, Jared Radtke, Sarah Zink, Previously Henry Erlich, Steven J. Mack, Anna Lisa Fear, Sandra Ke, Niveen Mulholland, Haitao Liu, John Nechtman, Yansheng Zhao, Na Jiang, Yanna Tian, Guangkuo Dong, Emily Farber, Rebecca Roche Pickin, Jonathan Davis, Jordan Davis, Dan Gallo, Jessica Bonnie, Paul Campolieto, Beena Akolkar, Kasia Bourcier, Thomas Briese, Suzanne Bennett Johnson, Eric Triplett

**Affiliations:** 1 Center for Public Health Genomics, University of Virginia, P.O. Box 800717, Charlottesville, VA 22908 USA; 2 Department of Public Health Sciences, University of Virginia, Charlottesville, VA 22908 USA; 3 Department of Biochemistry and Molecular Genetics, University of Virginia, Charlottesville, VA 22908 USA; 4 Anschutz Medical Campus, Barbara Davis Center for Childhood Diabetes, University of Colorado, Boulder, USA; 5 University of Turku, Turku, Finland; 6 Turku University Hospital, Hospital District of Southwest Finland, Turku, Finland; 7 Tampere University, Tampere, Finland; 8 Tampere University Hospital, Tampere, Finland; 9 National Institute for Health and Welfare, Helsinki, Finland; 10 University of Oulu, Oulu, Finland; 11 Oulu University Hospital, Oulu, Finland; 12 University of Kuopio, Kuopio, Finland; 13 Center for Biotechnology and Genomic Medicine, Augusta University, Augusta, USA; 14 University of Florida, Gainesville, USA; 15 Pediatric Endocrine Associates, Atlanta, USA; 16 Forschergruppe Diabetes E.V. and Institute of Diabetes Research, Helmholtz Zentrum München, Forschergruppe Diabetes, and Klinikum Rechts Der Isar, Technische Universität München, Munich, Germany; 17 Center for Regenerative Therapies, TU Dresden, Dresden, Germany; 18 Department of Nutritional Epidemiology, University of Bonn, Bonn, Germany; 19 Dr. Von Hauner Children’s Hospital, Department of Gastroenterology, Ludwig Maximillians University Munich, Munich, Germany; 20 Lund University, Lund, Sweden; 21 Pacific Northwest Research Institute, Seattle, USA; 22 Children’s Hospital of Pittsburgh of UPMC, Pittsburgh, USA; 23 University of South Florida, Tampa, USA; 24 Bristol Medical School, University of Bristol, Bristol, UK; 25 Center for Genetics, Children’s Hospital Oakland Research Institute, Oakland, USA; 26 NIDDK Biosample Repository at Fisher BioServices, Rockville, USA; 27 Jinfiniti Biotech, LLC, Augusta, USA; 28 National Institutes of Diabetes and Digestive and Kidney Diseases, Bethesda, USA; 29 National Institutes of Allergy and Infectious Diseases, Bethesda, USA; 30 Columbia University, New York, USA; 31 Florida State University, Tallahassee, USA

**Keywords:** Type 1 diabetes, Transcriptomics, Systems biology

## Abstract

Significant progress has been made in elucidating genetic risk factors influencing Type 1 diabetes (T1D); however, features other than genetic variants that initiate and/or accelerate islet autoimmunity that lead to the development of clinical T1D remain largely unknown. We hypothesized that genetic and environmental risk factors can both contribute to T1D through dynamic alterations of molecular interactions in physiologic networks. To test this hypothesis, we utilized longitudinal blood transcriptomic profiles in The Environmental Determinants of Diabetes in the Young (TEDDY) study to generate gene co-expression networks. In network modules that contain immune response genes associated with T1D, we observed highly dynamic differences in module connectivity in the 600 days (~ 2 years) preceding clinical diagnosis of T1D. Our results suggest that gene co-expression is highly plastic and that connectivity differences in T1D-associated immune system genes influence the timing and development of clinical disease.

## Introduction

Type 1 diabetes (T1D) is an autoimmune disease caused by the T cell-mediated destruction of insulin-producing β-cells in the pancreatic islets^1^. T1D is the most common chronic disease in children, with rapidly increasing rates, particularly in historically low prevalence populations^2^. T1D is typically preceded by islet autoimmunity, defined by persistence of at least one of three islet autoantibodies—insulin autoantibody (IAA), insulinoma antigen-2 autoantibody (IA-2A), or glutamic acid decarboxylase autoantibody (GADA). Recently, staging of disease progression has been defined by both the presence and number of autoantibodies^3^. Both islet autoimmunity and development of clinical T1D are influenced by genetic and environmental factors^4,5^. Identifying individual risk factors and how they interact is key to developing novel, personalized T1D prediction, intervention, and treatment strategies.

Recent studies suggest that complex diseases, such as T1D, are driven primarily by complex molecular networks whose function is altered by genetic and environmental risk factors^6^. Defining network topologies in T1D relevant cell-types represents a novel approach to understand how individual factors impact molecular networks in these cells and how network perturbations modify the risk of T1D. Gene co-expression network analysis, a method that groups genes based on similarity in expression profiles across a series of perturbations (*e.g*., intervention or genetic background), is an approach for constructing networks^7^. Gene co-expression networks have the advantage of providing information of how genes interact in specific cell types or tissues. Few studies have employed co-expression network studies of T1D^8,9^. The result of one study^8^ identified an interferon regulatory factor-7 (IRF7)-driven inflammatory network module associated with T1D risk. While prior studies have characterized risk factors and networks between prevalent T1D cases and controls, a major limitation of studies has been the use of gene expression profiles after disease onset with variable lengths of T1D duration (and treatment). Conceptually, gene expression should be obtained from pre-clinical subjects (those “at risk”) and followed over time. This optimal study design would provide information on the transitions in disease stages (from “at risk” to “disease initiation” to “disease progression” to “disease onset”) and the changes in network topology, thereby providing insight on the features leading from risk to islet autoimmunity and, ultimately, T1D.

To better understand the dynamic nature of co-expression networks and their role in T1D, we examined gene expression from participants in The Environmental Determinants of Diabetes in the Young (TEDDY) study^9^. The primary aim of TEDDY is to identify factors influencing the development of islet autoimmunity and T1D through the long-term monitoring of children at high genetic (based upon HLA genotype) risk. From an initial cohort of over 8000 children, TEDDY has obtained samples at multiple time points prior to development of islet autoimmunity and T1D.

Here, we used weighted gene co-expression network analysis (WGCNA) to define the dynamic nature of co-expression networks preceding progression to T1D^10,11^. We observed that the majority of modules in a reference network exhibited differences that distinguish those TEDDY participants that develop T1D (and becoming ‘cases’) from those not progressing (‘controls’) during at least one period in the 600 days preceding T1D diagnosis. Two of the modules were enriched for genes associated with T1D that play key roles in immune function, suggesting a possible causal contribution to islet autoimmunity, progression rate, and/or development of T1D.

## Results

### Construction of a reference T1D co-expression network

**Figure 1:**
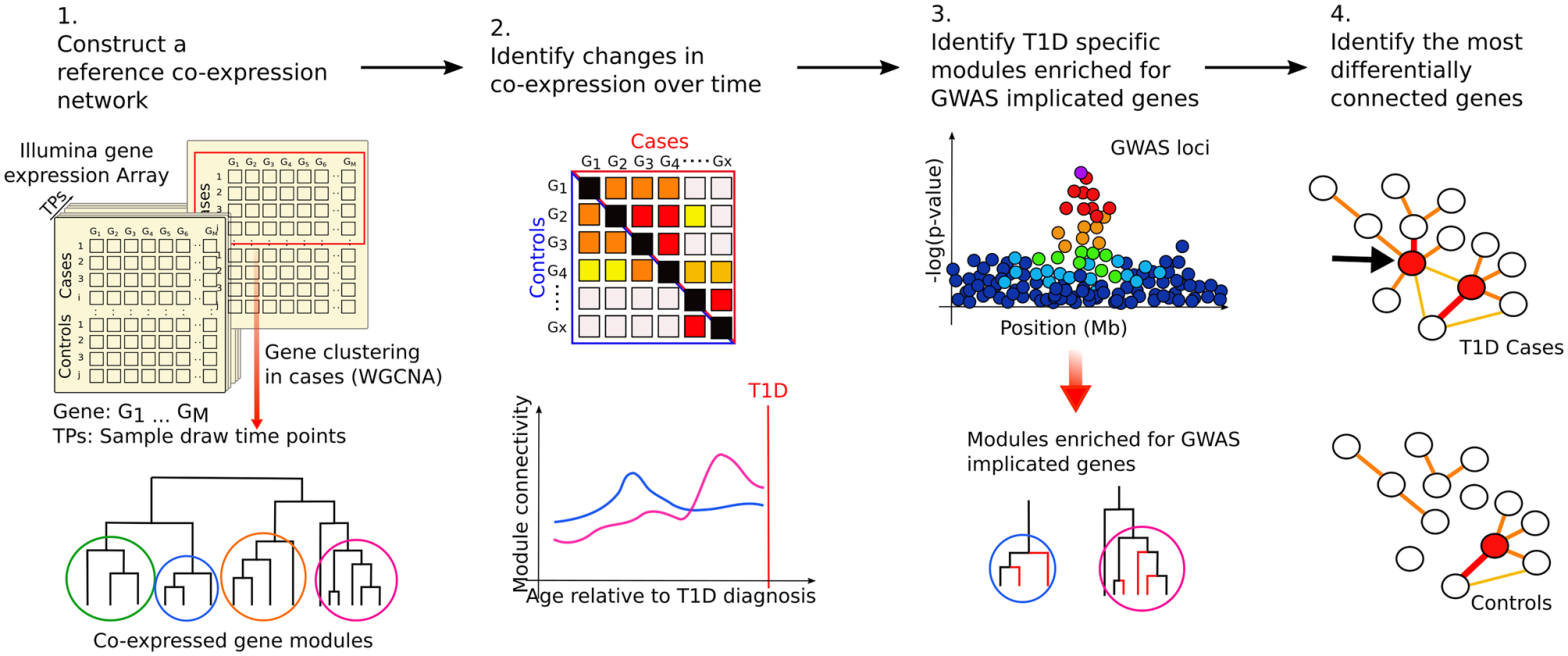
Construction of a “reference” T1D co-expression network enabled the exploration of changes of gene network dynamics across time, sex and disease status. (1) TEDDY transcriptomic profiles were grouped based on time from diagnosis and a “reference” WGCNA network was constructed for cases sampled approximately 180 days before T1D diagnosis. (2) Reference network modules were tested for enrichment of GWAS implicated genes. (3) For modules identified in step 2, we evaluated longitudinal differences in connectivity between cases and controls, (4) and we investigated the role of individual genes module behavior and T1D.

The conceptual framework of the analytic process is shown in Fig. 1. Initially, TEDDY whole blood transcriptomic profiles (N = 1921) were grouped into 10-day intervals, starting at 600 days before diagnosis through progression to islet autoimmunity and T1D onset. This strategy enables the identification of network differences specific to T1D independent of differences in age at diagnosis. A “reference” network was created using WGCNA^11^ and gene expression profiles from those TEDDY participants who progressed to T1D (N = 60), collected 180 days prior to date of diagnosis. This reference network enabled comparisons of network dynamics across time, sex, and disease status. The reference network contained 25,528 genes partitioned into 36 co-expression modules with module sizes ranging from 19 genes to 1823 genes (Supplementary Table 1).

### Dynamic changes in module connectivity between T1D cases and controls

**Figure 2:**
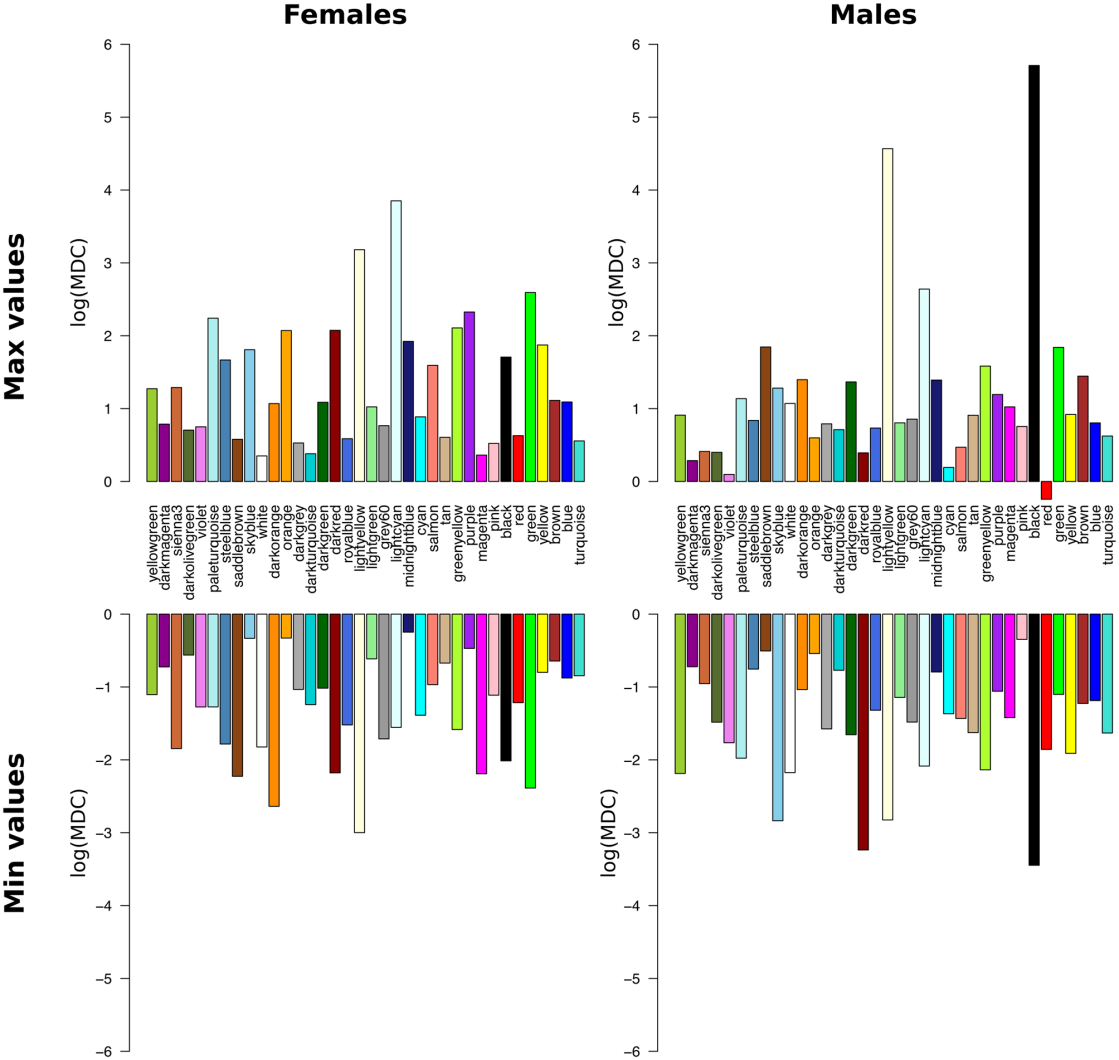
Maximum and minimum logarithmic MDC values in females and males over a time period of 600 days per module. The modules are ordered by module size.

TEDDY provides unique longitudinal gene expression profiles; thus, a key question is whether global network topology (*i.e.*, organization of gene–gene relationships) differs between those who develop T1D (cases) and controls (those who do not during that time period). A secondary question is whether the network topologies (gene–gene relationships) change over time. To characterize longitudinal change, module connectivity (sum of all pairwise gene connections in a module) was calculated for all modules in the reference network for each 10-day interval. Modular Differential Connectivity (MDC), defined as the ratio of whole module connectivity in T1D cases relative to controls, was then calculated^12^. Across the 600-days preceding T1D diagnosis, all 36 modules displayed significantly (*P* < 0.05) increased or decreased MDC during at least one 10-day interval in either males or females (with log(MDC) values ranging from -3 to 3.9 in females and -3.4 to 5.7 in males; Fig. 2 and Supplementary Fig. 1). Although MDC values and patterns differed by sex, the mean change in MDC over time was not significantly (*P* > 0.05) different between males and females.

### Identification of “immune” modules enriched for T1D GWAS genes

**Figure 3:**
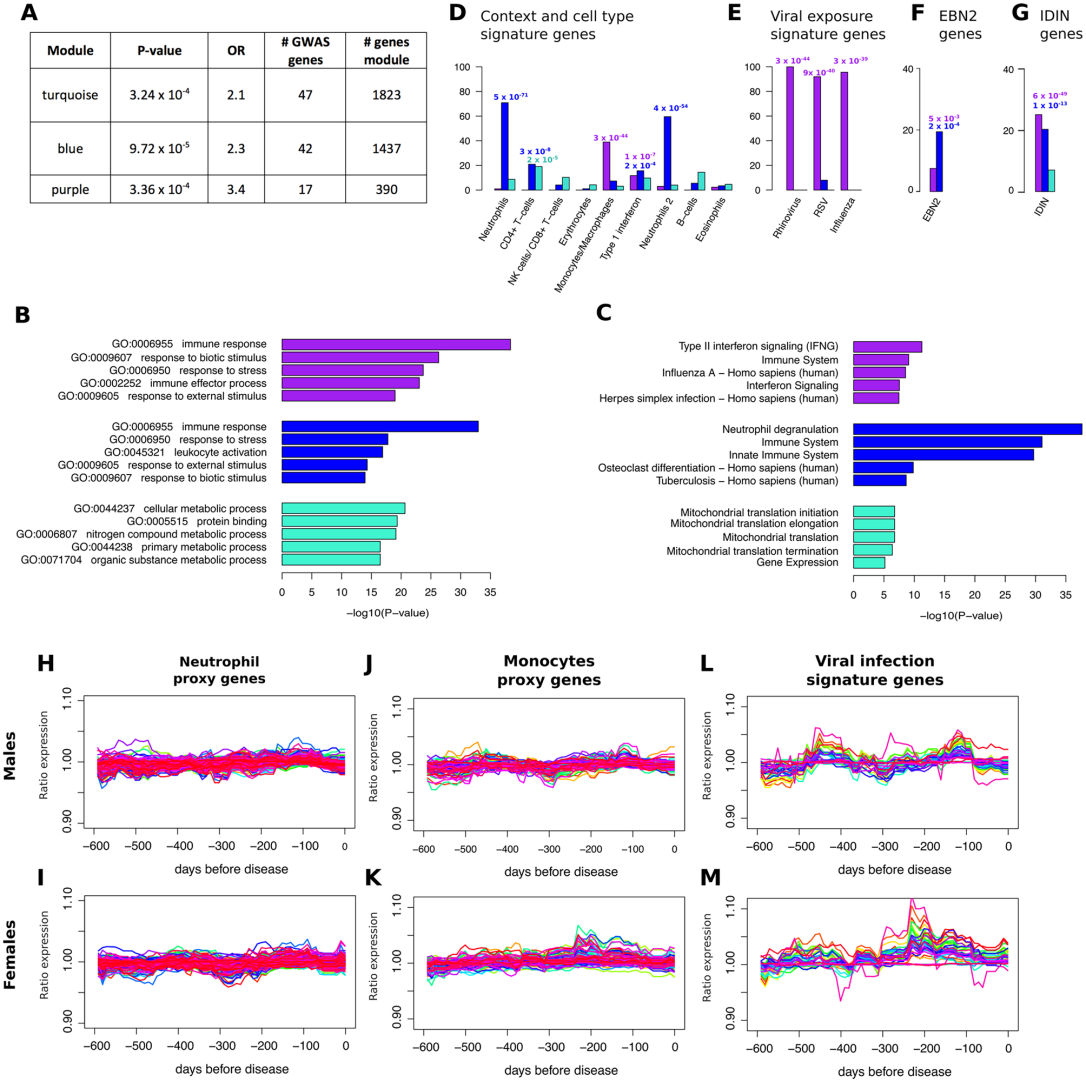
Characterization of the TEDDY blood co-expression network. (**A**) Modules enriched for T1D GWAS-implicated genes (adjusted using FDR method). (**B**, **C**) GO term and pathway enrichment based on ConsensusPathDB^15^. The P values are FDR adjusted P values. (**D**–**G**) All enrichment P values are adjusted based on FDR method (**D**) Percentage of context dependent signature genes found in modules based on proxy genes identified by Zhernakova et al.^16^ (**E**) Percentage of genes differentially expressed under viral infection identified by Zaas et al.^17^ (**F**) Percentage of genes differentially expressed between EBV + and EBV- Ramos B cells^18^ (**G**) Percentage of genes found in the IRF7-driven inflammation network based on Heinig et al.^9^ (**H**–**K**) Ratio of expression in cases versus controls over time for cell type proxy genes (**L**, **M**) Ratio of expression in cases versus controls of viral infection signature genes .

Based upon TEDDY blood transcriptomic data, MDC appears to be highly dynamic over time. To focus on those changes directly impacting T1D risk, we identified modules containing genes with an increased likelihood of being causally associated with risk of developing T1D. Prior studies have demonstrated that co-expression modules are more likely to be causally related to a disease if they are enriched for genes implicated by genome-wide association studies (GWAS)^7,13^. Using data from robustly-defined T1D-associated GWAS results^14^, we identified genes implicated by GWAS and mapped them onto the reference network (Supplementary Table 2). The purple (FDR = 3.36 × 10^–4^), blue (FDR = 9.72 × 10^–5^), and turquoise (FDR = 3.24 × 10^–4^) modules were significantly enriched for T1D GWAS-implicated genes (Fig. 3A).

To further prioritize the three modules enriched for T1D GWAS-implicated genes, genes relevant to immune function were identified. Gene ontology (GO) enrichment analyses revealed that the purple and blue modules were enriched for immune-related GO terms and pathways, including the terms “immune response” (purple module FDR = 1.0 × 10^–33^; blue module FDR = 4.9 × 10^–41^) and “immune system” (purple module FDR = 8 × 10^–10^; blue module FDR = 8 × 10^–32^) (Fig. 3B, C and Supplementary Table 3). In contrast, the top GO term enrichments for the turquoise module were not immune-related. The top turquois module GO term was “cellular metabolic process” (FDR = 2.2 × 10^–21^) (Fig. 3B, C and Supplementary Table 3). Based on the enrichment of immune related genes, the blue and the purple modules were the focus of subsequent analyses.

The purple and blue modules were enriched for genes representative of specific immune cell types^16^. The purple module was enriched for genes in the monocyte/macrophage signature gene set (FDR = 2 × 10^–44^) while the blue module was enriched for two different sets of neutrophil signatures genes (with gene overlaps of 71% and 60%, FDR = 5 × 10^–71^ and FDR = 4 × 10^–54^, respectively) (Fig. 3D).

The purple module contained genes reported to be upregulated during Rhinovirus infection (Fig. 3E)^17^, as well as being enriched for genes upregulated by influenza and respiratory syncytial virus (RSV) infections (FDR ranged from 3 × 10^–39^ to 3 × 10^–44^) (Fig. 3E). Epstein Barr Virus (EBV) protein has been associated with autoimmune diseases^18^, and both the purple and the blue modules were enriched for genes differentially expressed between EBV + and EBV- Ramos B cells (FDR = 5 × 10^–3^ and 2 × 10^–4^) (Fig. 3F). Both purple (FDR = 6 × 10^–49^) and blue (FDR = 1 × 10^–13^) modules were enriched for genes in an interferon regulatory factor 7 (IRF7)-driven inflammatory network, also previously associated with T1D^9^ (Fig. 3G).

Co-expression within the purple and blue modules could be due to correlations induced by changes in the proportions of immune cell-types and not by intrinsic cellular connections between genes. The expression of the monocyte/macrophage and neutrophil immune cell-type signature genes^16^ was compared in T1D cases and controls. Only three genes (*SUSD1*, *GBA* and *MFSD7*) had significant (*P* < 0.05) differential expression in either males or females. No gene exceeded a 10% difference in gene expression between T1D cases and controls (Fig. 3H–K), and none of the viral infection signature genes were differentially expressed at any time point (Fig. 3L, M).

### Dynamic changes in purple and blue module connectivity between T1D cases and controls

**Figure 4:**
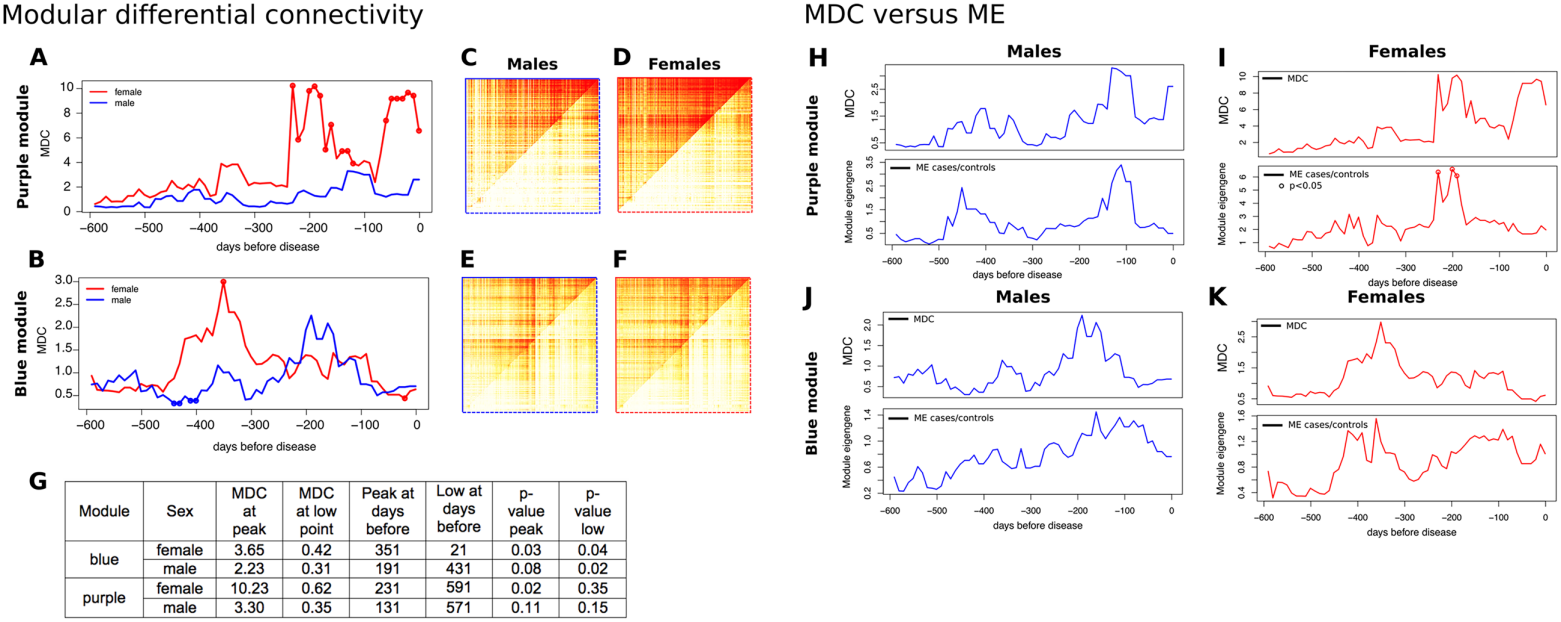
Dynamic changes in connectivity in the blue and purple modules. (**A**) and (**B**) module differential connectivity (MDC) over time in intervals of 10 days for the purple and blue modules, respectively. Solid points indicate a significant difference between cases and controls (FDR < 0.05). (**C**–**F**) Direct comparison of topological overlap matrix for cases (upper left triangle) and controls (lower right triangle) at the MDC peak time point. (**G**) MDC metrics. (**H**, **I**) Change in MDC in the purple module correlates with change in the module eigengene (ME). MDC and ME patterns were strikingly similar, except for the last time point before diagnosis in the purple module. The correlated patterns suggest that gene expression influences, MDC but is not the only factor. Solid points indicate a significant difference between cases and controls (*P* < 0.05). (**J**, **K**) Change in MDC in the blue module also correlates with change in ME.

Modular Differential Connectivity (MDC) was highly dynamic over time for both blue and purple modules and both sexes (Fig. 4A–F). In the purple module, there was a greater than tenfold increase in connectivity in females starting at 231 days before T1D diagnosis, with two primary peaks of high MDC occurring at 231 days (FDR *P* = 0.02) and at 21 days (FDR *P* = 0.002). Two peaks were observed in males, at 131 days and 11 days, although the changes were not significant. In the blue module, the MDC in females was significantly increased at 351 days before T1D diagnosis and connectivity was increased by more than threefold in cases (FDR *P* = 0.03), and the MDC was significantly lower at 21 days (FDR *P* = 0.04). In males, MDC was increased (not significantly) at 191 days, but was significantly lower between 450 and 400 days (FDR *P* = 0.02) before T1D diagnosis Fig. 4G.

The enrichment of the purple module for genes involved in the response to viral infections suggested that module connectivity might be influenced by season. Although there was no clear seasonal MDC pattern in the 600-day time-frame, control samples were partitioned into four groups representing the four seasons and compared connectivity. Despite seasonal variance, connectivity in controls was lower than the values observed at the MDC peak in cases suggesting that season is not responsible for the differences in MDC (Supplementary Fig. 2). In addition, MDC was not correlated with uneven seasonal distribution of cases and controls (Supplementary Fig. 2). Although seasonal patterns have been observed for the incidence rate of T1D^19^, these data suggest that changes in MDC are not driven by season.

### Subtle differences in module gene expression partly underlie changes in connectivity

Differences in gene expression were assessed between T1D cases and controls over time for all blue and purple module genes. Of 2229 module probes (1802 genes), only three genes (*RILPL1, IL15RA and KIAA08952*) were differentially expressed (FDR < 0.05). Module eigengene (ME) was used to capture gene expression at the entire module level and differential ME (DME) was used as a measure of differential modular expression between T1D cases and controls. DME displayed a similar pattern to that seen for MDC, where greater gene expression was associated with higher connectivity (though DME was significant [FDR < 0.05] only for females in the purple module) (Fig. 4H–K). These patterns were observed for both blue and purple modules in both sexes. The MDC in the purple module showed a marked increase within three weeks of development of T1D (21 days in females and 11 days in males), with no increase in DME during this time. These data suggest that longitudinal changes in module connectivity were largely due to subtle, but highly coordinated, changes in the aggregate expression of module genes.

### Identifying genes driving dynamic changes in MDC

**Figure 5:**
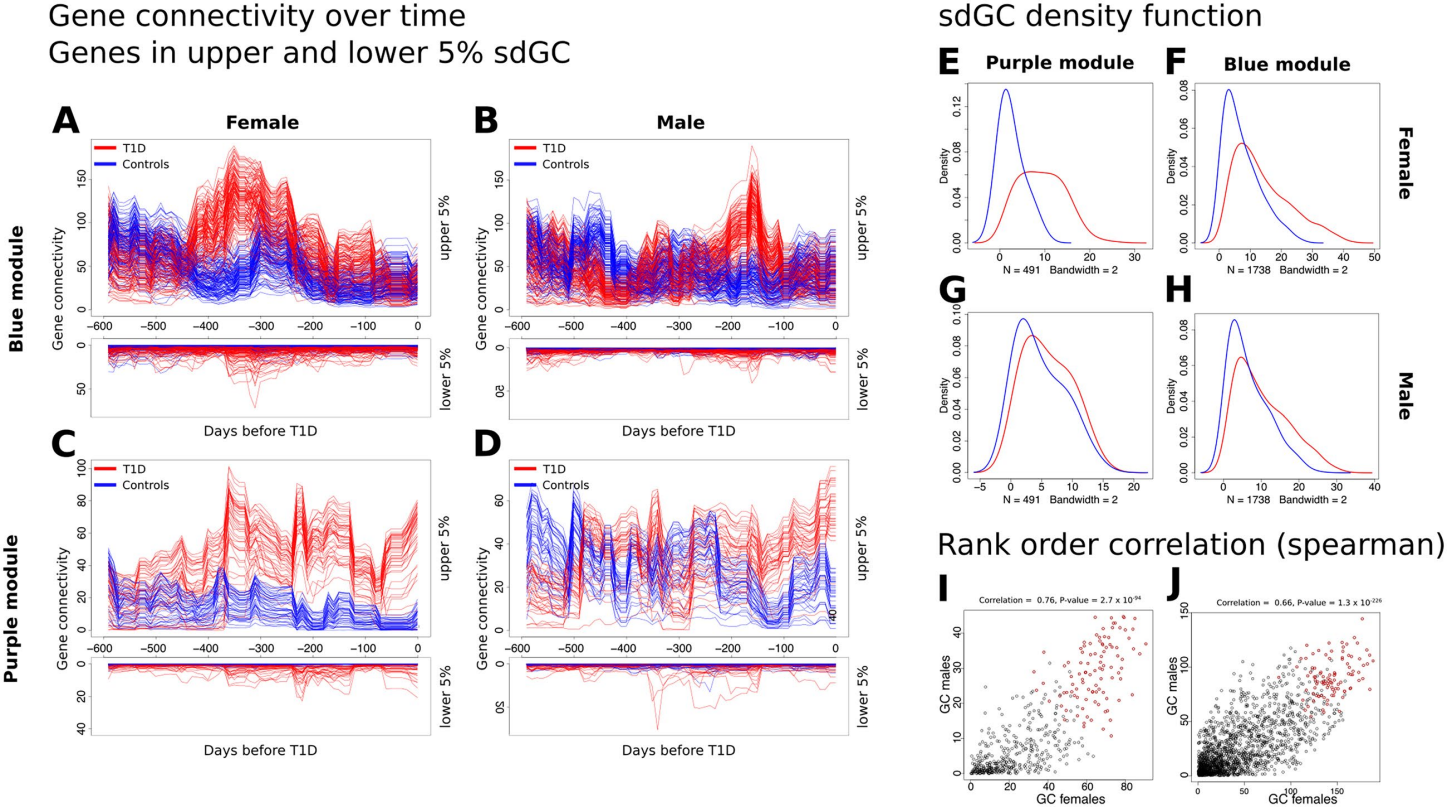
Assessing gene connectivity in Blue and Purple module. (**A**–**D**) Gene connectivity (GC) over time. Red: cases, blue: controls. Genes were ranked based on variance in connectivity (based on standard deviation over time). Upper plot shows the upper 5 percent of genes based on GC for cases and controls. Lower plot shows genes of the lower 5 percentile of genes based on GC in cases and controls. (**E**–**H**) Density function of the standard deviation of the GC over time. We observed a shift to the right in cases (red line) compared to controls (blue line) that was more profound in females (**E**, **F**) than males (**G**, **H**). (**C**) Rank order correlation (Spearman correlation) of GC at the MDC peak between females (**I**) and males (**J**). Red dots show the top 100 differential hub genes.

The temporal pattern of gene connectivity for genes with the largest changes in connectivity mimicked the MDC patterns (Fig. 5A–H). However, many genes demonstrated minor changes in connectivity. Hub genes, the most highly connected genes in a network, play important roles in biological networks^20^ and disease^21,22^. Given the difference in the temporal pattern of MDC between the sexes in both modules (Fig. 3A, B), the same set or different sets of genes were evaluated as drivers of MDC differences between sexes. The rank-order correlation between males and females for gene connectivity was 0.66 (*P* = 1.3 × 10^–226^) for the blue module and 0.76 (*P* = 2.7 × 10^–94^) for the purple module (Fig. 5I, J). The high correlation suggests that the same genes were involved in connectivity changes in both sexes, even though the timing of these changes differed. The most differentially connected genes (DCGs) were identified by subtracting gene connectivity in controls from T1D cases and their ranks summed across the sexes to obtain a final ranked list. The top 100 DCGs (Fig. 5I, J depicted in red) are hubs in their original modules, consistent with changes in MDC primarily driven by hub genes. The top 10 DCGs for each module are listed in Table 1 (Supplementary Table 4 and Supplementary Table 5 contains the complete ranked gene list for purple and blue module respectively).

**Table 1:** Top 10 differentially connected genes for blue and purple modules.

Module	DCG (ordered by rank)	Infection/cell type signature gene*	Differential connectivity (peak) females	Differential connectivity (peak) males	Differential connectivity p value (MDC peak) females	Differential connectivity p value (MDC peak) males
Blue	*TLR8*		3.65	5.18	0.02	0.01
	*KCNJ15*	Neutrophils	3.89	6.76	0.02	0.02
	*FCGR2A*		3.09	4.97	0.04	0.05
	*BEST1*	Neutrophils	3.99	3.34	0.04	0.07
	*UBR2*		5.29	5.67	0.03	0.03
	*ALPK1*		3.14	4.54	0.05	0.03
	*PGCP*		6.62	4.27	0.01	0.05
	*FPR2*		4.92	3.75	0.02	0.03
	*PLBD1*		7.76	6.34	3 × 10^–3^	0.02
	*MXD1*	Neutrophils	2.81	3.25	0.05	0.03
Purple	*SAMD9*		16.52	3.76	6 × 10^–3^	0.06
	*MX2*		9.90	6.52	0.03	0.04
	*CMPK2*	Monocytes	14.12	7.56	0.04	0.03
	*SIGLEC1*	Infection	1726.99	12.76	2 × 10^–3^	0.03
	*USP18*		30.03	8.99	2 × 10^–3^	0.04
	*IFIH1*		15.25	3.47	6 × 10^–3^	0.06
	*DDX58*	Infection	8.46	9.08	0.03	0.02
	*OASL*	Infection	8.30	4.37	0.13	0.05
	*CXCL10*	Infection	37.82	11.90	9 × 10^–3^	0.04
	*USP41*		1738.34	16.86	< 1 × 10^–4^	0.06

*Cell type proxy genes identified by Zhernakova et al.^16^ or viral infection signature genes identified by Zaas et al.^17^ Differential Connectivity was calculated by dividing connectivity in cases by controls and significance was calculated by permutations.

### Large increases in connectivity for DCGs with links to T1D

**Figure 6:**
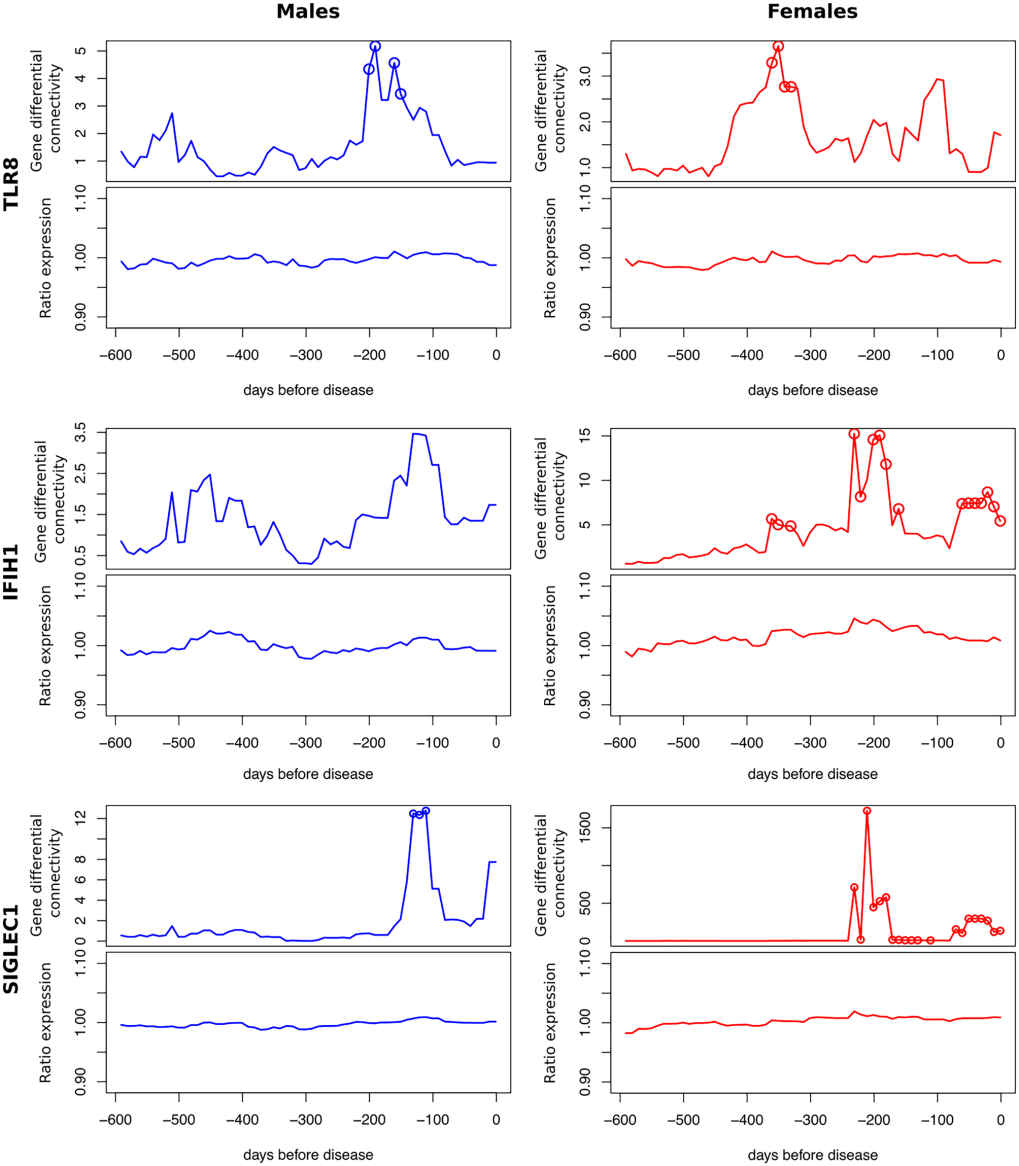
Gene connectivity (upper) and gene expression (lower) for *TLR8* (blue module) and *IFIH1* and *SIGLEC1* (purple module). Gene differential connectivity *P* values < 0.05 are shown as circles.

Three of the top DCGs (Table 1) are Toll-like receptor 8 (*TLR8*) from the blue module, and Sialic Acid Binding Immunoglobulin Like Lectin 1 (*SIGLEC1*) and Interferon induced with helicase C domain 1 (*IFIH1*) from the purple module (Table 1). These three genes have significantly (*P* < 0.05) increased GDC values that reflect the MDC changes observed in their respective modules (Fig. 6). The ratio of gene expression in T1D cases relative to controls was determined for each gene. In contrast to the large differences in connectivity, only subtle increases (< 5%) in expression between T1D cases and controls were observed. The timing of the increased GDC and expression were consistent for each gene.

## Discussion

Over the last decade, significant progress has been made in elucidating genetic factors impacting T1D, yet we know little of how genetic and environmental inputs converge on molecular networks and how network alterations influence T1D risk. We reconstructed whole blood gene co-expression networks in TEDDY using longitudinal data in at-risk participants with observed conversion to islet autoimmunity and clinical T1D. The analysis of module connectivity as a function of time revealed widespread differences in connectivity between those who progressed to T1D (cases) and those who did not (controls). Modules of co-expressed immune response genes with strong genetic association with T1D were identified. These critical modules demonstrated dynamic alterations in connectivity preceding T1D diagnosis. Module-level changes in connectivity appeared to be driven by module hub genes, with most changes in connectivity at the gene-level were due to subtle differences in gene expression. These data, for the first time, demonstrate the plasticity of longitudinal gene co-expression relationships, highlight the limitations of disease transcriptomics studies investigating disease at a single time point after diagnosis, and inform our understanding of T1D.

In this study, we observed network-wide changes in connectivity between T1D cases and controls over time. Though we did not know what to expect a priori, our results are in line with other network studies of disease. For example, Zhang et al.^12^ compared MDC in brain co-expression network modules between late-onset Alzheimer’s disease patients and controls. They found that over half (54%) of identified modules had higher connectivity in cases compared to controls, at a single time-point. In addition, 4.5% had lower connectivity in cases compared to controls. Therefore, it is not surprising that in our study MDC differed between cases and controls during at least one time-point for all network modules. Because of the longitudinal nature of our data, we were able to rigorously compare the relationship between differential expression and MDC. We observed that most, but not all, of the differences in MDC were likely due to subtle changes (< 5%) in expression between cases and controls. This suggests that connectivity “amplifies” small, otherwise undetectable, changes in gene expression. This amplification effect likely underlies the widespread network changes we observed in MDC. It also demonstrates that potentially important biological changes in network homeostasis occur independent of detectable changes in expression.

One challenge with co-expression network analysis (along with all transcriptomics and other “-omic” studies) is establishing causality. Do network changes influence disease or are they just responding to a difference in disease state? This was especially key for this study given that changes in MDC did little to highlight modules of interest. To address this limitation, we identified modules that were enriched for GWAS genes^13^. We and others have shown that such modules are more likely to be causally related to disease^7,13^. Hence, the strong genetic link between the two modules identified and T1D suggest these modules capture changes that have a direct role in driving the initiation of islet autoimmunity or progression to T1D.

Interestingly, we observed that the timing and magnitude of changes in connectivity, but not the participating genes, differed between males and females in the purple and blue modules. Sex differences are well documented for auto-immune diseases, where females, in general, have stronger immune responses^23^. For instance, interferon response and neutrophil percentage is higher in females than in males, in the context of disease^23,24^. Whereas some of the sex differences are influenced by sex hormones, differences in pediatric cases are thought to be due to genetic differences^23^. Compared to other auto-immune diseases, there is not a strong female bias in T1D incidence^25^. However, in TEDDY, the progression rate after multiple islet autoantibodies was found to be higher in females than in males^26^. In our study, overall connectivity in the purple and blue modules was higher in females and the peak in connectivity for both modules occurred earlier. Although our results support the idea that the same mechanisms underlie T1D development and progression in males and females, the response to genetic and environmental risk factors may differ and cause the difference observed in timing of connectivity increases before T1D.

Our analysis of the purple and blue modules suggest they represent the activity of distinct immune cell types. The purple module was enriched for interferon signaling genes, viral infection signature genes, differentially expressed genes in EBV infected B-cell and monocyte/macrophage proxy genes. The purple module genes also overlap genes of an IRF7-driven inflammation network (IDIN) that was derived from isolated monocytes^9^. Increased interferon signaling preceding T1D diagnosis has been reportedx^27^. However, the mechanisms by which interferon signaling alters the immune response and T1D remain elusive. A recent study found that type I interferon potentiates β-cell-reactive CD8 + T-Cell cytotoxicity^26^. In addition, type I interferon promotes inflammatory monocytes and helps sustain chronic inflammation^28^. This suggests, that while interferon signaling allows for effective clearance of viral infections, it might potentiate inflammation within the islet cells. These results are consistent with previous studies reporting a potential association between viral infections and T1D^29^.

The blue module was enriched for neutrophil signature genes and genes differentially expressed in EBV infected B-cells. It was also enriched for IDIN genes, although not as strong as the purple module. Neutrophils direct and interact with many immune cells such as macrophages and dendritic cells. They both regulate the innate and the adaptive immune responses^30–32^ and are thought to play an important role in T1D and other autoimmune diseases^30,33^. Neutrophils are believed to perform a large number of roles in autoimmunity, including antigen presentation, activation of T and B cells and direct tissue damage^31,33^. In addition, reduced neutrophil levels have been associated with increased risk of T1D^34,35^.

Due to the scale-free properties of co-expression networks, networks consist of a few highly connected genes (hub genes) and a large number of lowly connected genes. In most networks, hub genes are thought to fundamentally determine the behavior of networks and have been shown to play important roles in disease^36–39^. We demonstrated that genes whose connectivity changes the most over time between cases and controls were often hub genes. If the perturbations observed here do truly impact T1D then it is likely that the identified hubs play a major role in T1D development and progression. We identified genes with known roles in T1D such as *IFIH1* among the list of differentially connected genes in the purple and blue modules. *IFIH1*, which encodes MDA5 (Melanoma Differentiation-Associated protein 5), binds viral dsRNA and initiates type I and type III interferon response. Variants in *IFIH1* have been associated with T1D and other autoimmune diseases^9,40^, and have been linked to increased basal and ligand triggered interferon I response^41^. In addition to *IFIH1*, genes such as *TLR8*, functions in activation of innate immunity and mediates production of cytokines, and *SIGLEC1,* functions in pathways related to innate immune system and antigen presentation, were also found to be differentially connected genes. TLR and Siglec family members are known to function in immune tolerance^42,43^ and therefore may also play an important role in progression to T1D.

Our study does have limitations. Although, T1D is a heterogeneous disease^44,45^ the TEDDY design generated a relatively homogenous group. We sorted samples based on the offset to T1D diagnosis, assuming that the rate of progression is approximately the same for all T1D cases. Our rationale for this was that all TEDDY subjects have high-risk HLA haplotypes and in this study all cases developed T1D at relatively young age (mean diagnosis age is 3 years). Additionally, because we were only able to track changes in connectivity over a time period of 600 days, half of the cases were taken after seroconversion. This only allows us to identify factors associated with progression of T1D, rather than autoimmunity. To look into factors associated with initiation of islet autoimmunity, we would need to limit the analysis to cases that were collected prior to seroconversion; however, statistical limitations due to the smaller size of the subgroup limited our ability to establish stable networks and therefore restricted us from making biological insights into factors that play a role in initiation of islet autoimmunity. Due to the unique TEDDY study design, we were not able to validate our findings in an independent cohort. Hence, caution should be practiced in generalizing our results beyond the scope of this study, especially regarding seroconversion and selected HLA subgroups.

In conclusion, we observed differences in the connectivity of immune function genes with genetic links to T1D that preceded disease development. These changes were highly dynamic as a function of time before T1D diagnosis. Our data suggest that gene–gene relationships are much more plastic than previously appreciated, which has important implications for network and transcriptomic studies of disease. These results also increase our understanding of the molecular networks and genes influencing T1D.

## Methods

### TEDDY design

The TEDDY study is a prospective cohort study funded by the National Institutes of Health^46^. The primary goal is to identify environmental causes of type 1 diabetes. TEDDY includes six clinical research centers—three in the US: Colorado, Georgia/Florida, Washington and three in Europe: Finland, Germany, and Sweden. A detailed study design has been previously described. For all study participants, written informed consents were obtained from a parent or primary caretaker, separately, for genetic screening and participation in prospective follow-up. The present study was approved by local U.S. Institutional Review Boards and European Ethics Committee Boards and is monitored by an External Evaluation Committee formed by the National Institutes of Health. All methods were carried out in accordance with relevant guidelines and regulations.

### Gene expression data

Gene expression was measured using the Illumina HumanHT-12 v4.0 expression BeadChip. Quality assessments were performed by boxplots of intensity values, plotting control probes and estimating the proportion of expressed genes in samples. The BASH method^47^ was used for beads artifact detection, which takes local spatial information into account when determining outliers. Background correction and normalization processes was performed to reduce differences due to technical variation while conserving true biological effects.

We filtered the data set based on population structure using principal component analysis. We removed all individuals of non-European ancestry. In addition, we calculated pairwise correlation for all samples and removed samples with mean correlation less than 0.95.

The final cohort consisted of 385 children including 60 T1D cases (30 males/ 30 females) and 325 T1D negative controls (171 males/ 154 females). In total, the dataset contains 1921 samples, with an average number of 6.7 samples per individual in cases and 4.7 in controls. The oldest children were 6 years of age.

For our time course analysis, we grouped samples based on their offset in days to T1D diagnosis. Specifically, we focused on a timeframe starting 600 days before and ending at T1D diagnosis. In steps of 10 days, we identified the sample from each individual collected closest to this particular time point. We only included samples that were drawn with an offset of less than 100 days. For each sample at each time-point, we identified a control sample that was matched regarding age and gender. A control was defined as an individual not diagnosed with T1D and without persistent confirmed autoantibody. We generated two sets of controls and compared cases with the average in controls. At the start of our analysis time frame, 30 of 60 T1D samples were taken after seroconversion (13 males, 17 females). See Supplementary Table 6 for details regarding age at seroconversion, age at diagnosis and gender and Supplementary Fig. 3 for sample distribution at each time-point.

### GWAS implicated genes

We identified genes implicated by GWAS using the latest GWAS fine mapping on T1D^14^. We annotated all 2021 credible T1D SNPs (46 loci) using the annotation database SCAN (http://www.scandb.org/). For each SNP, we identified the host gene, the right and left flanking genes and potential eQTL genes (*P* < 1 × 10^–4^ in CEU). This approach led to a total of 444 GWAS implicated genes (Supplementary Table 2).

### Differential expression and module eigengene

We used a two-sided t-test to estimate statistical significance of differential expression. We adjusted the p value threshold based on Bonferroni correction (0.05/number of tests, *i.e.* probes).

As a measure of module expression, we computed the module eigengene (ME), which is the first principal component (PC) calculated via principal component analysis (PCA). For this we used the R library “prcomp”. The rotation matrix (matrix of the loadings, *i.e*. eigenvectors) was computed using the reference network, for each module respectively. For each time point, we calculated the ME values by rotating the expression data using the PCA rotation matrix of the reference network. By using the same rotation, we ensured that the same ME was used for all time-points. As for differential gene expression, t-test was used to estimate difference between ME values in cases and controls.

### Weighted gene co-expression network analysis

Network analysis was performed using the WGCNA R package^11^. We selected a power threshold of 12 using the scale-free topology criterion. Modules (groups of co-expressed genes) are found by average linkage hierarchical clustering, which uses the topological overlap measure as dissimilarity. To achieve the clustering, we chose a cut off height of 0.25. Because of the high number of genes, we used the blockwiseModules function with a maximum block size of 20,000 and a minimum module size of 30.

### Modular connectivity and gene connectivity

As a measure of gene co-regulation, we calculated the connectivity for each gene and module. Gene wise, the connectivity is defined as the sum of connection strengths with the other module genes: ${k}_{i}= {\sum }_{u\ne i}{a}_{ui}$, where ${a}_{ij}$ is the correlation to the power of 8 between gene i and j. The gene connectivity (GC) is defined as: $G{C}_{i}= {\sum }_{j=1}^{N}{k}_{ij}$. We used the topological overlap matrix (as described by Langefelder and Hovath^11^) instead of adjacency matrix for modular connectivity (MC). ${t}_{ij}$ is calculated as:

$${t}_{ij}= \left\{\begin{array}{c}\frac{{l}_{ij}+{a}_{ij}}{min\left\{{k}_{i},{k}_{j}\right\}+1-{a}_{ij}} \\ 1\end{array}\genfrac{}{}{0pt}{}{if i\ne j}{if i=j}\right.$$

where ${l}_{ij}= {\sum }_{u}{a}_{iu}{a}_{{u}_{j}}, k={\sum }_{u}{a}_{iu}$ and the index u runs across all nodes of the network.

MC is calculated by: $MC= {\sum }_{i=1}^{N-1}{\sum }_{j=i+1}^{N}{t}_{ij}$ and the modular differential connectivity is:

$$MDC\left(x,y\right)= \frac{{\sum }_{i=1}^{N-1}{\sum }_{j=i+1}^{N}{t}_{ij}^{x}}{{\sum }_{i=1}^{N-1}{\sum }_{j=i+1}^{N}{t}_{ij}^{y}}$$

Significance was calculated through false discovery rate, with 1000 permutations (M). We differentiate two scenarios, gain of connectivity (MDC > 1) and loss of connectivity (MDC < 1):

$$FD{R}_{MDC>1}= \frac{1}{M} \sum_{p=1}^{M}MDC\left(x, y\right)>MDC({x}_{p},{y}_{p})$$

$$FD{R}_{MDC<1}= \frac{1}{M} \sum_{p=1}^{M}MDC\left(x,y\right)<MDC({x}_{p},{y}_{p})$$

### Differential hub genes and differential connected genes

To identify genes highly connected in cases but not highly connected in controls (differential hub genes; DHG), we subtracted GC in controls from GC in cases: $\delta {H}_{i}=G{C}_{{i}_{cases}}- G{C}_{{i}_{controls}}$. To quantify the difference between cases and controls, we calculated the differential connectivity. Analogue to MDC, differential gene connectivity (GDC) was defined as the ratio between GC in cases and controls:

$$GDC=G{C}_{{i}_{cases}}/G{C}_{{i}_{controls}} .$$

Significance was estimated through false discovery rate (as done for MDC), with 1000 permutations.

## Supplementary Information

Supplementary Figure 1.

Supplementary Figure 2.

Supplementary Figure 3.

Supplementary Table 1.

Supplementary Table 2.

Supplementary Table 3.

Supplementary Table 4.

Supplementary Table 5.

Supplementary Table 6.

## Data Availability

Gene Expression data have been deposited in NCBI’s database of Genotypes and Phenotypes (dbGaP) with the primary accession code phs001562.v1.p1.
