# Supplementary material for: Dynamic changes in immune gene co-expression networks predict development of type 1 diabetes
Source: Sci Rep. 2021 Nov 22;11:22651. doi: 10.1038/s41598-021-01840-z (PMC8609030; doi:10.1038/s41598-021-01840-z)
Supplement: Supplementary file 1 — Supplementary Figure 1. [file 41598_2021_1840_MOESM1_ESM.pdf]

Supplementary Figure 1: Dynamic changes in connectivity for all modules. Module Differential Connectivity (MDC) over time in intervals of 10 days. Solid points indicate a significant difference between cases and controls (FDR<0.05). MDC in females is shown in red, MDC in males is shown in blue.

pink MDC over time (cases/ctr)

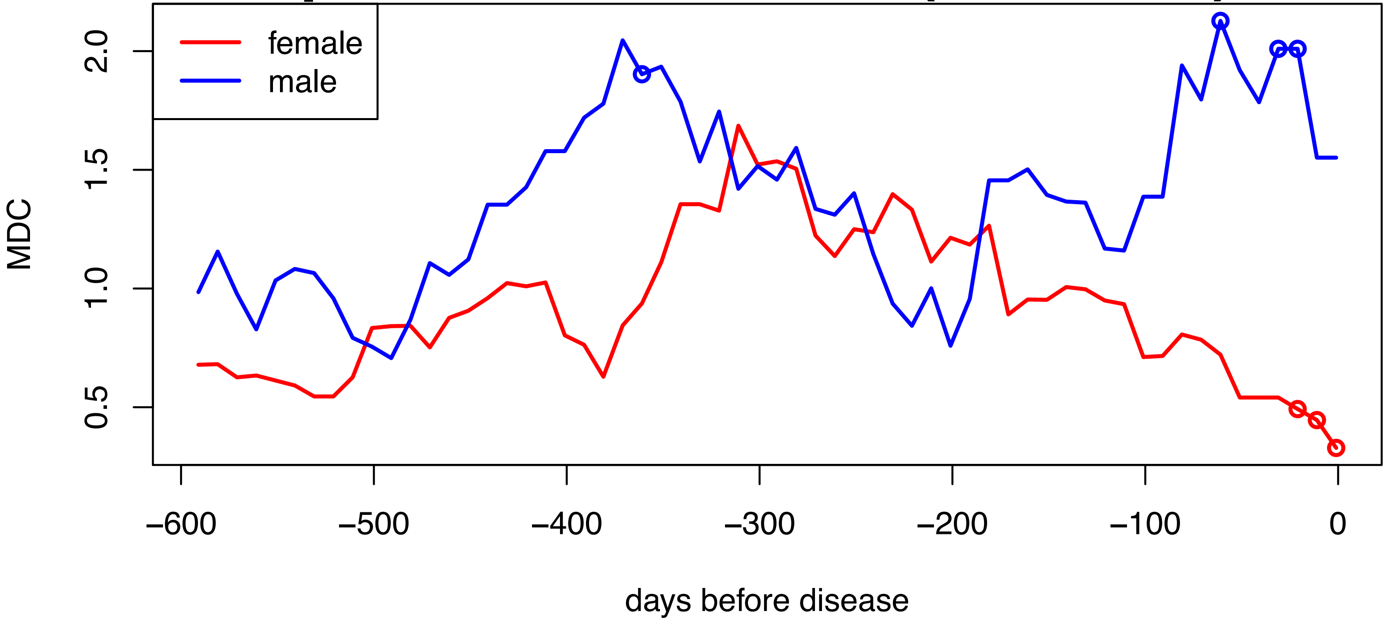

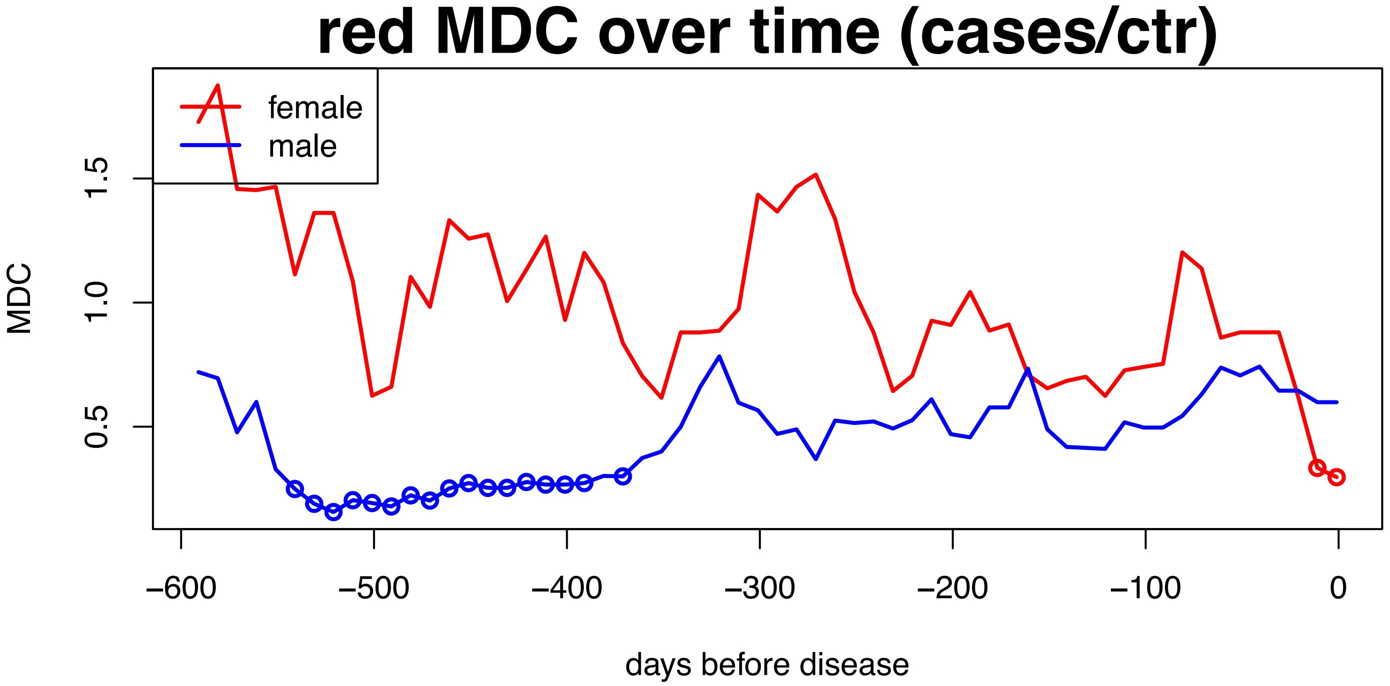
red MDC over time (cases/ctr)
female
male
MDC
0.5
1.0
1.5
−600
−500
−400
−300
−200
−100
0
days before disease

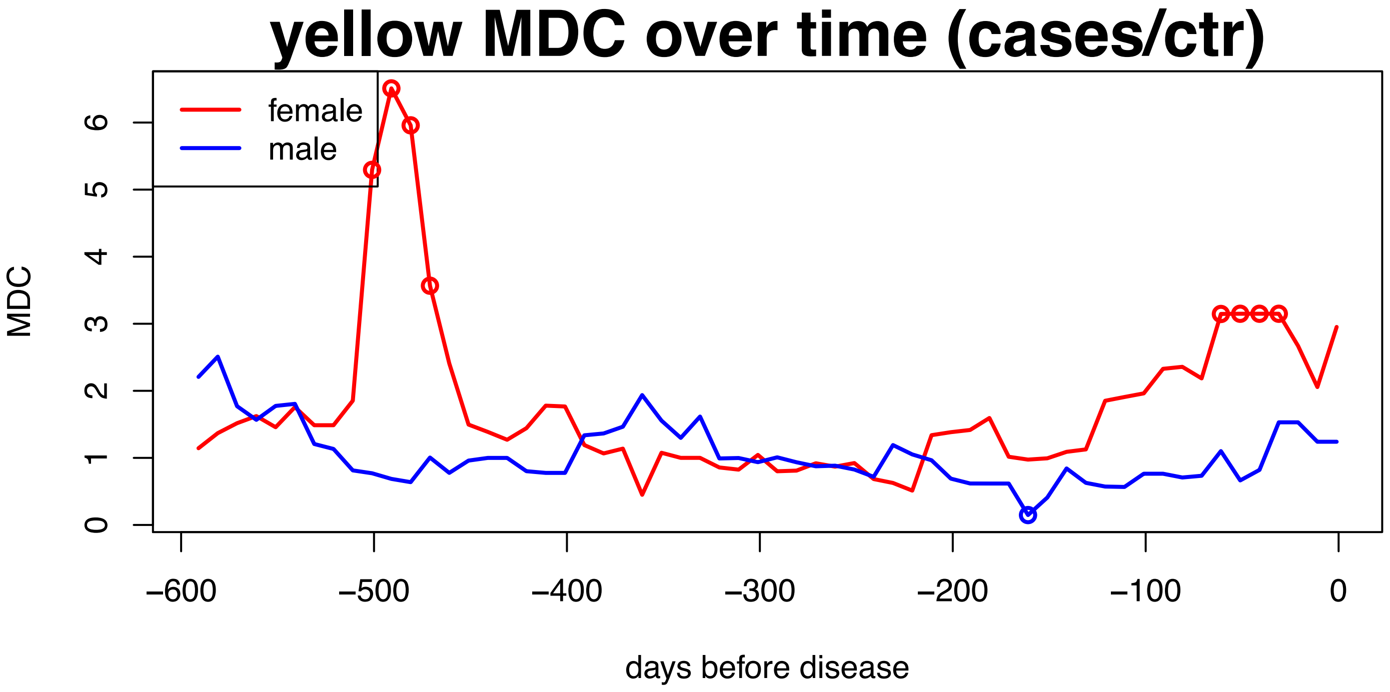
yellow MDC over time (cases/ctr)
female
male
MDC
0
1
2
3
4
5
6
−600
−500
−400
−300
−200
−100
0
days before disease

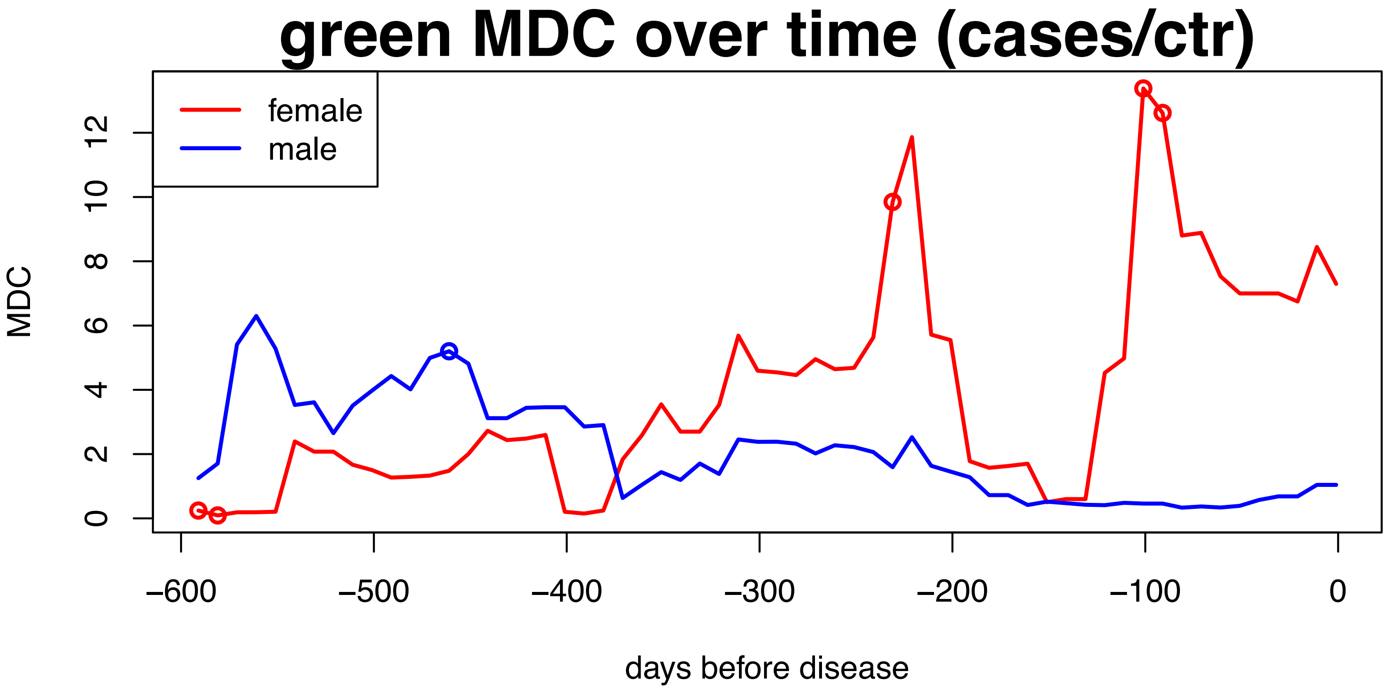

green MDC over time (cases/ctr)
female
male
MDC
0
2
4
6
8
10
12
−600
−500
−400
−300
−200
−100
0
days before disease

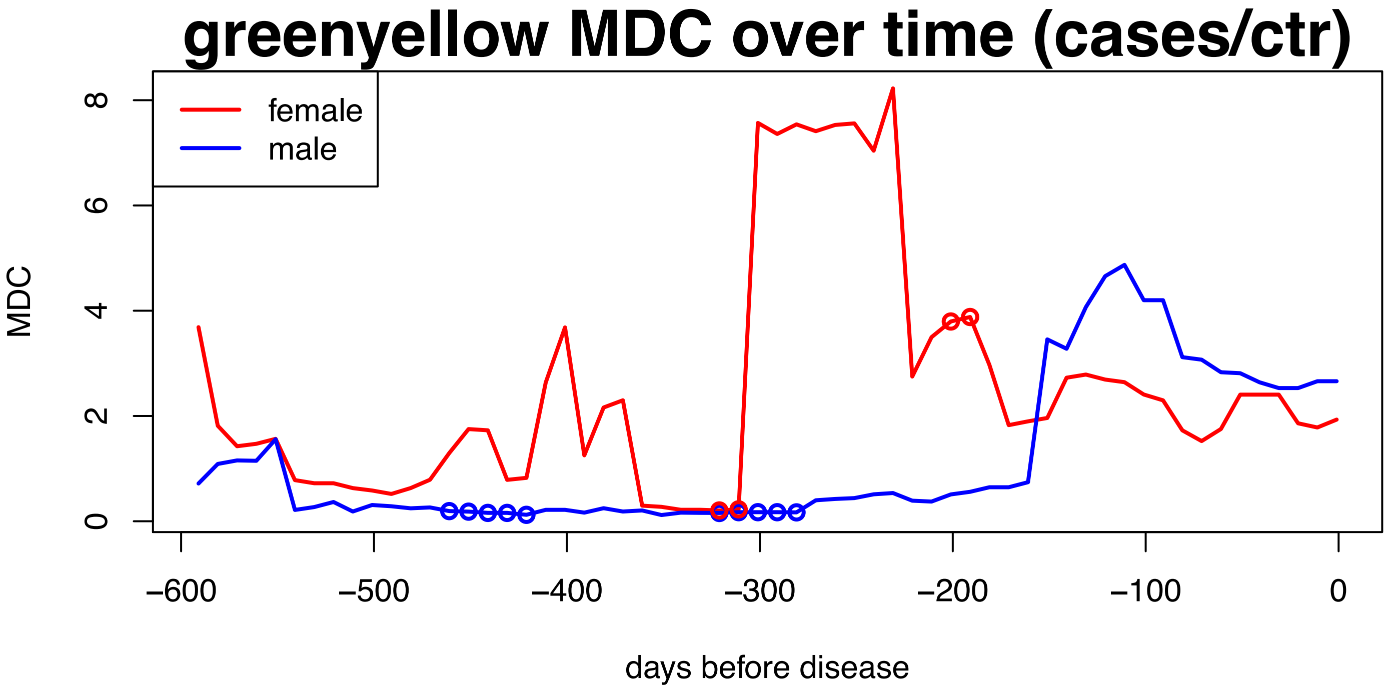

greenyellow MDC over time (cases/ctr)
female
male
MDC
0
2
4
6
8
−600
−500
−400
−300
−200
−100
0
days before disease

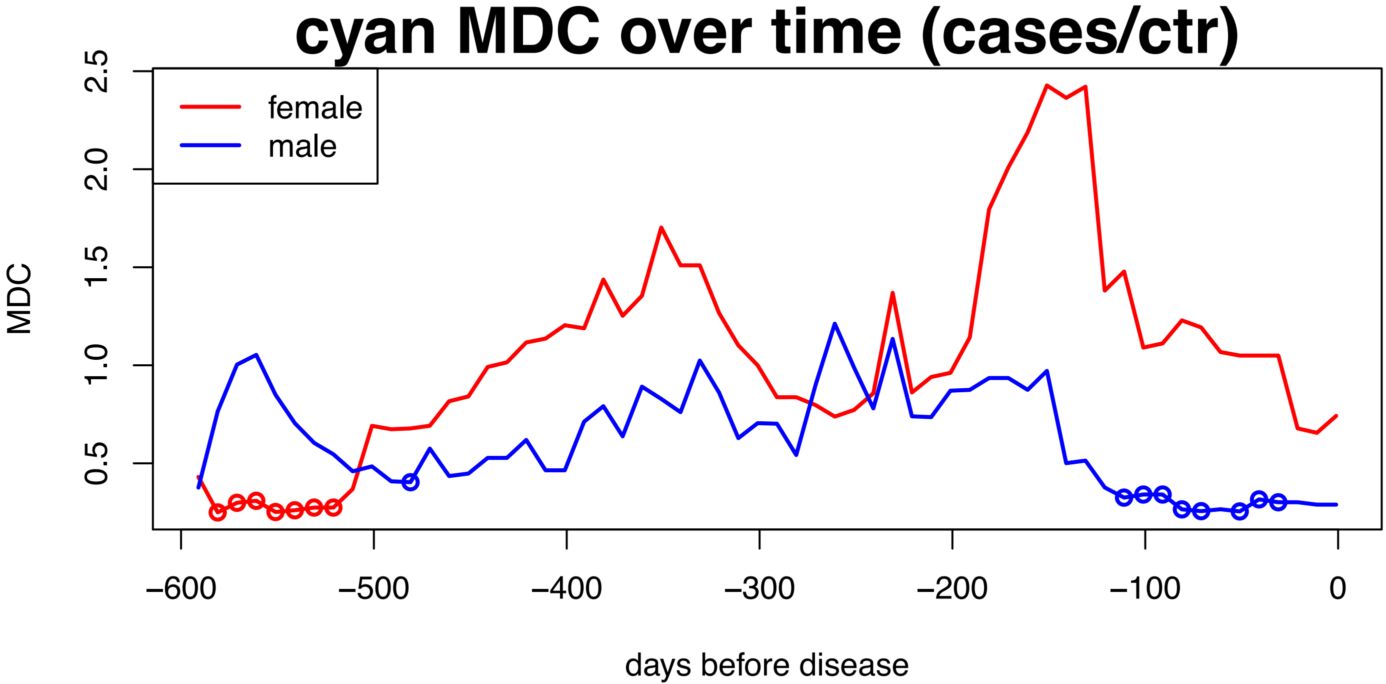
cyan MDC over time (cases/ctr)
female
male
MDC
0.5
1.0
1.5
2.0
2.5
−600
−500
−400
−300
−200
−100
0
days before disease

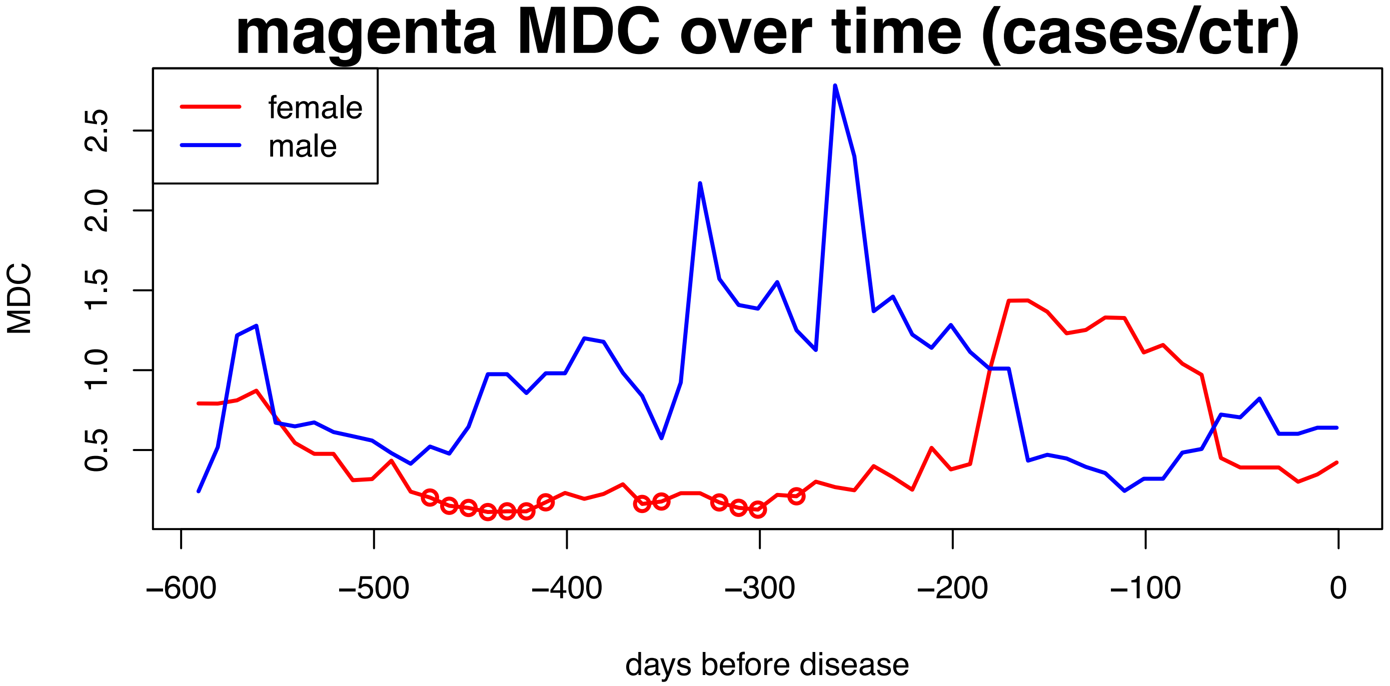
magenta MDC over time (cases/ctr)
female
male
MDC
0.5
1.0
1.5
2.0
2.5
−600
−500
−400
−300
−200
−100
0
days before disease

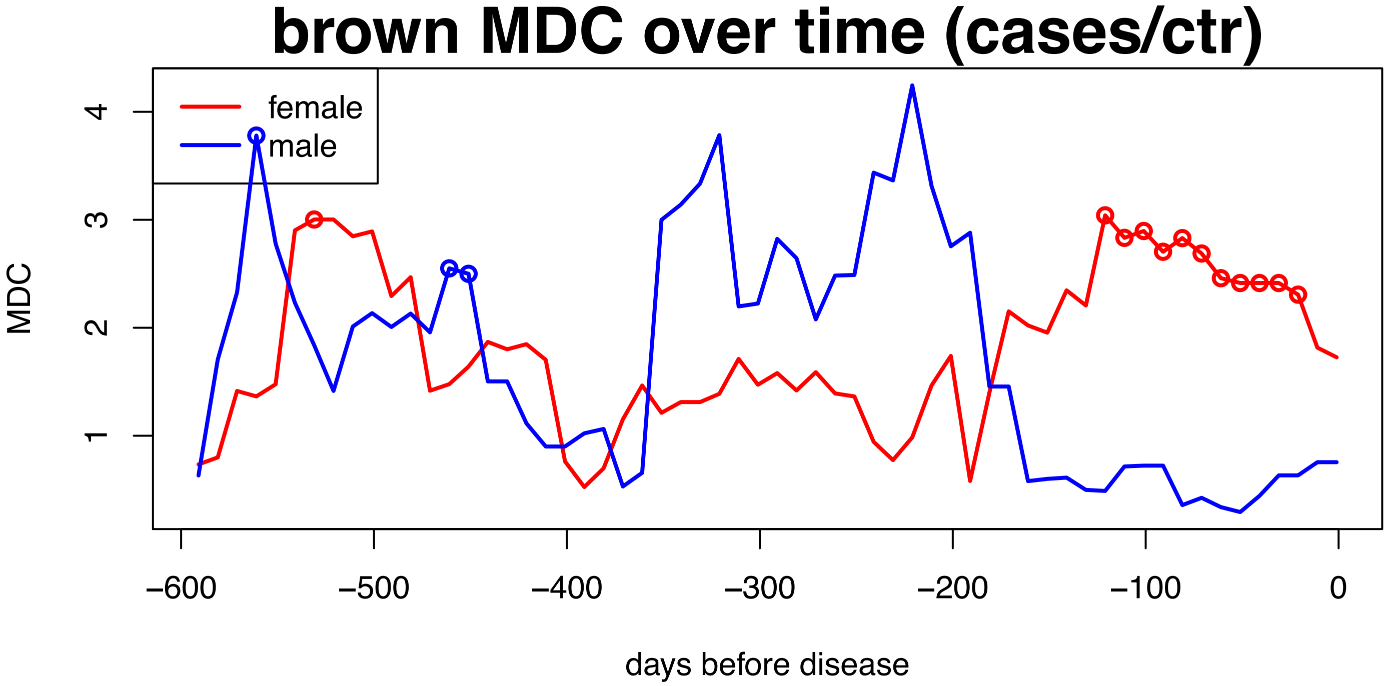

brown MDC over time (cases/ctr)
female
male
MDC
4
3
2
1
−600
−500
−400
−300
−200
−100
0
days before disease

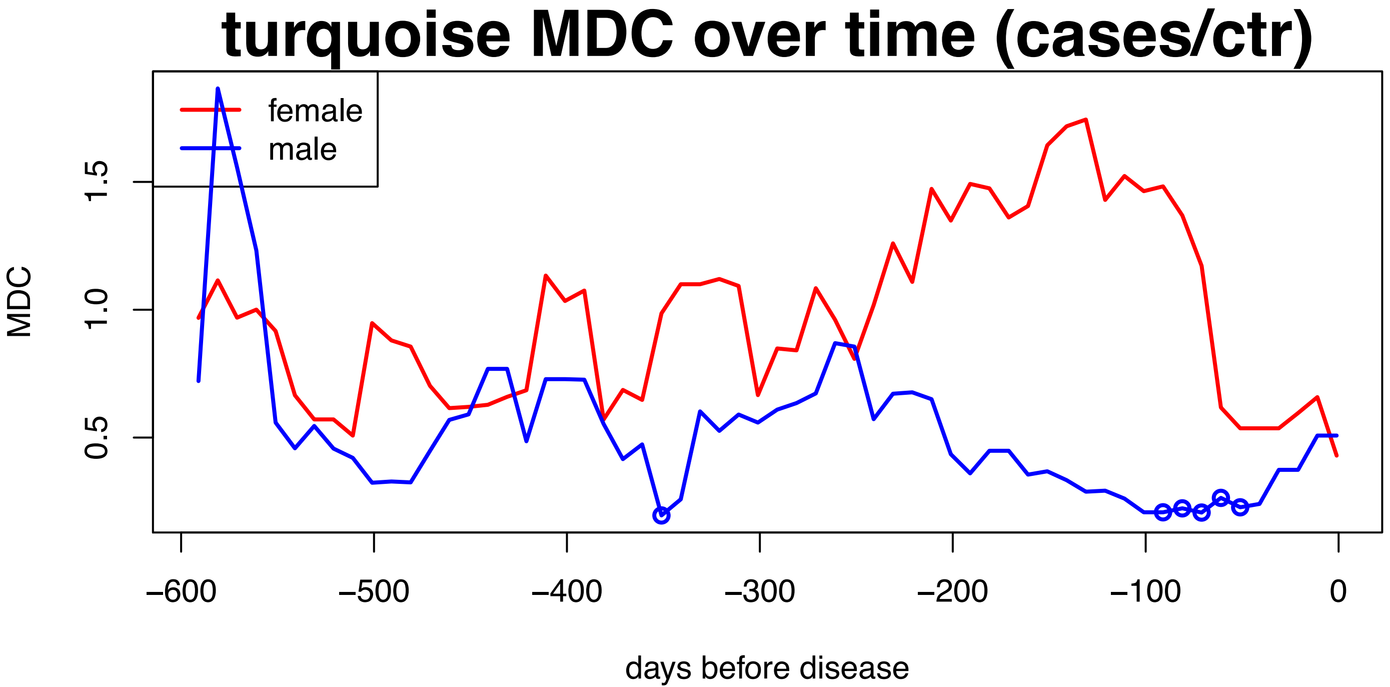
turquoise MDC over time (cases/ctr)
female
male
MDC
0.5
1.0
1.5
−600
−500
−400
−300
−200
−100
0
days before disease

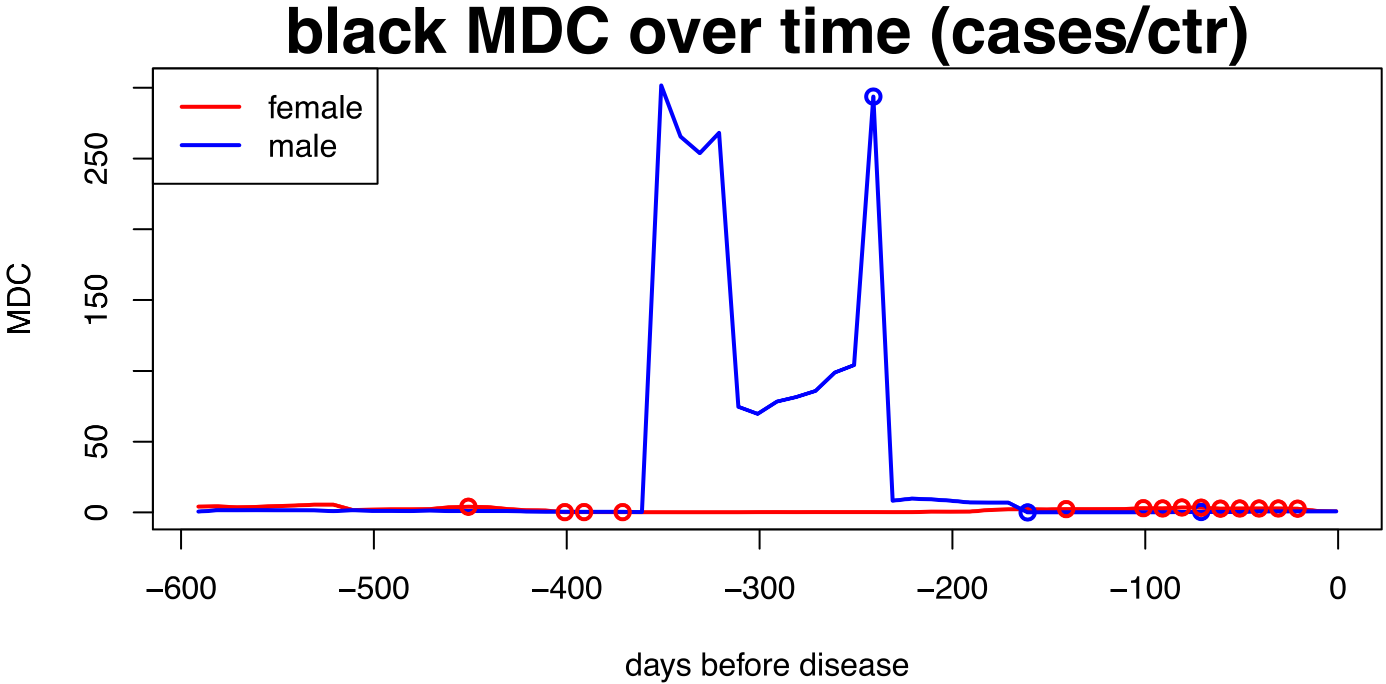

black MDC over time (cases/ctr)
female
male
MDC
0
50
150
250
–600
–500
–400
–300
–200
–100
0
days before disease

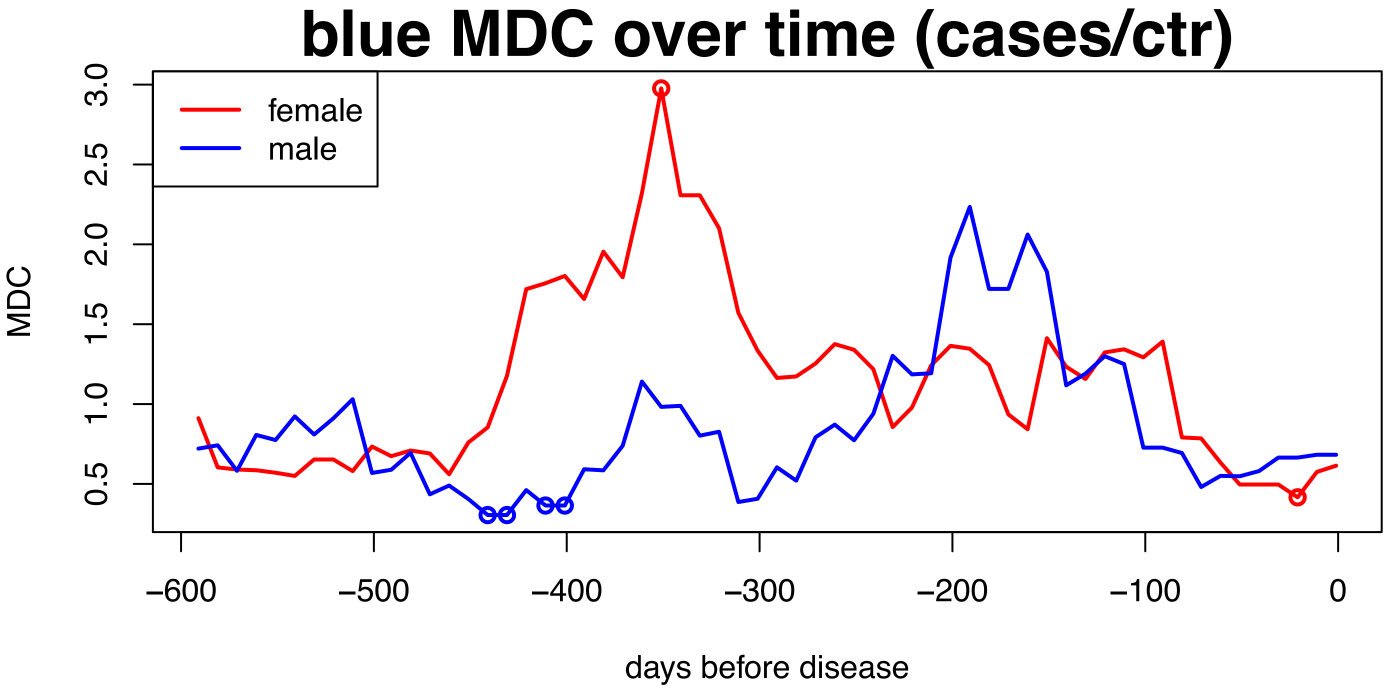

blue MDC over time (cases/ctr)
female
male
MDC
0.5
1.0
1.5
2.0
2.5
3.0
−600
−500
−400
−300
−200
−100
0
days before disease

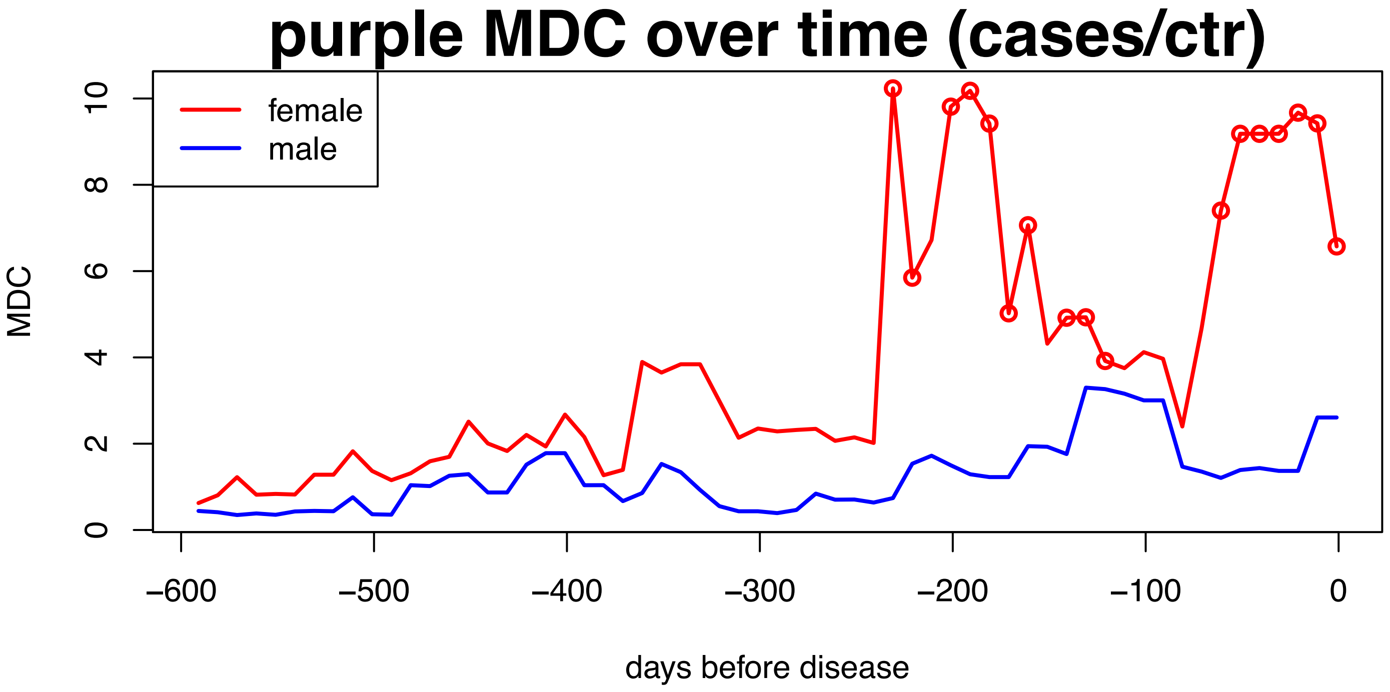
purple MDC over time (cases/ctr)
female
male
MDC
0
2
4
6
8
10
−600
−500
−400
−300
−200
−100
0
days before disease

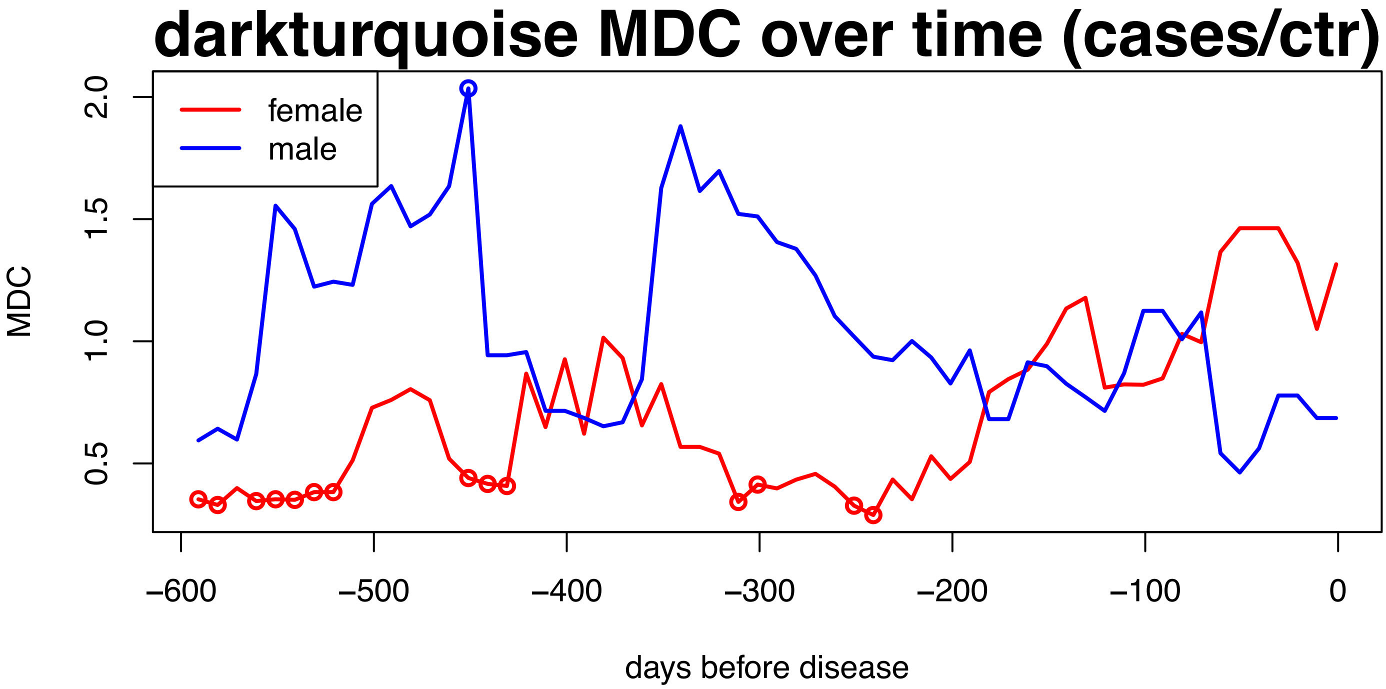

darkturquoise MDC over time (cases/ctr)
female
male
MDC
2.0
1.5
1.0
0.5
−600
−500
−400
−300
−200
−100
0
days before disease

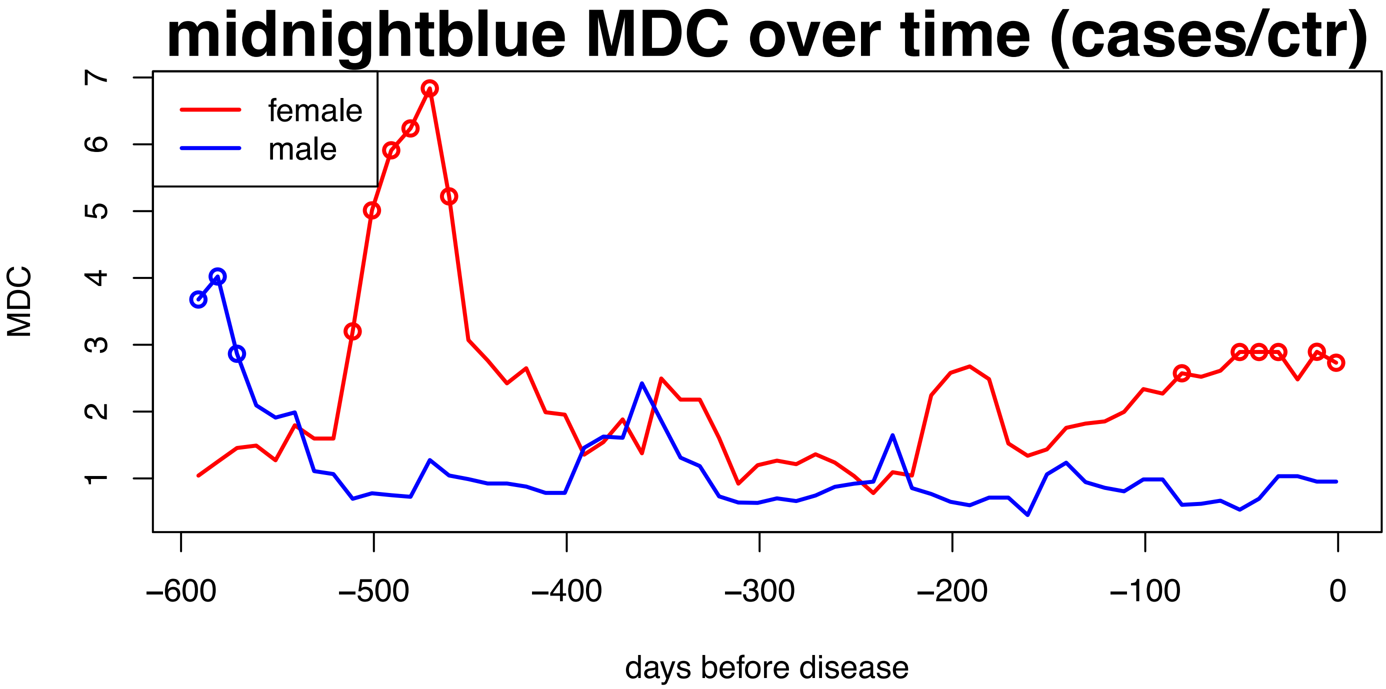

midnightblue MDC over time (cases/ctr)
female
male
MDC
1
2
3
4
5
6
7
−600
−500
−400
−300
−200
−100
0
days before disease

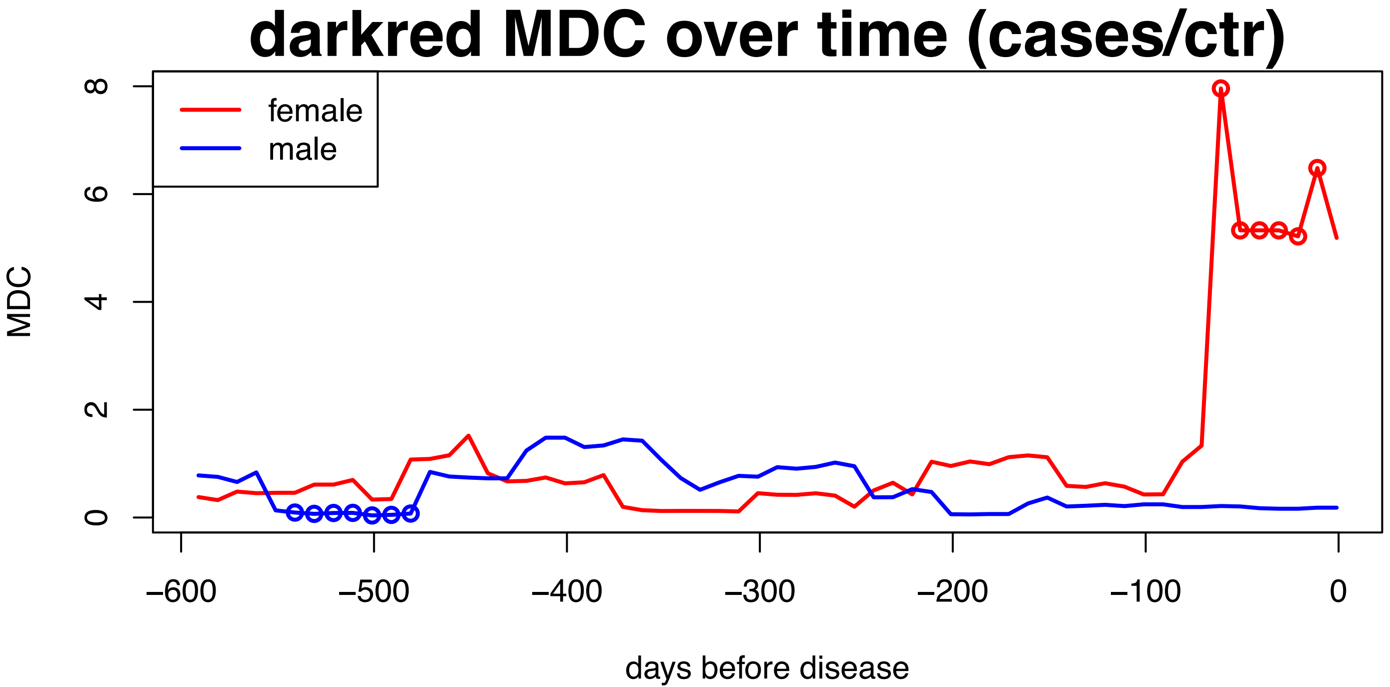
darkred MDC over time (cases/ctr)
female
male
MDC
0
2
4
6
8
−600
−500
−400
−300
−200
−100
0
days before disease

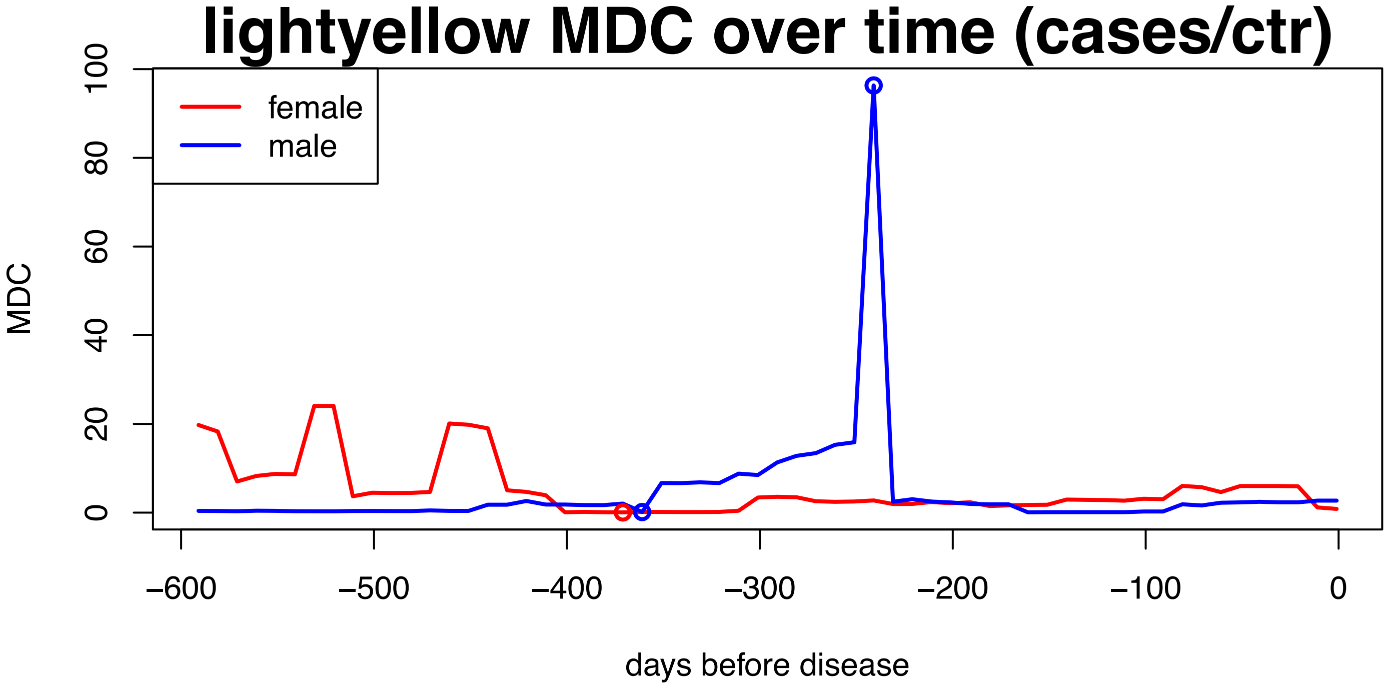

lightyellow MDC over time (cases/ctr)
female
male
MDC
0
20
40
60
80
100
−600
−500
−400
−300
−200
−100
0
days before disease

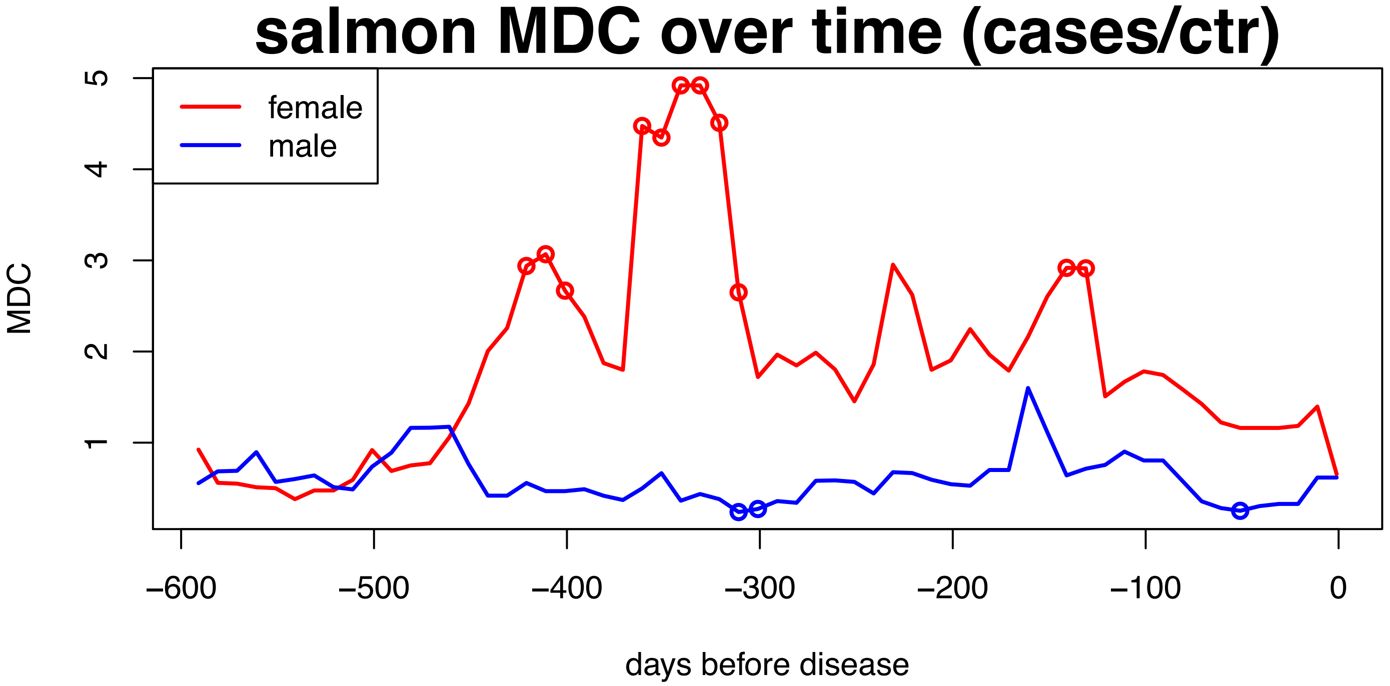
salmon MDC over time (cases/ctr)
female
male
MDC
5
4
3
2
1
−600
−500
−400
−300
−200
−100
0
days before disease

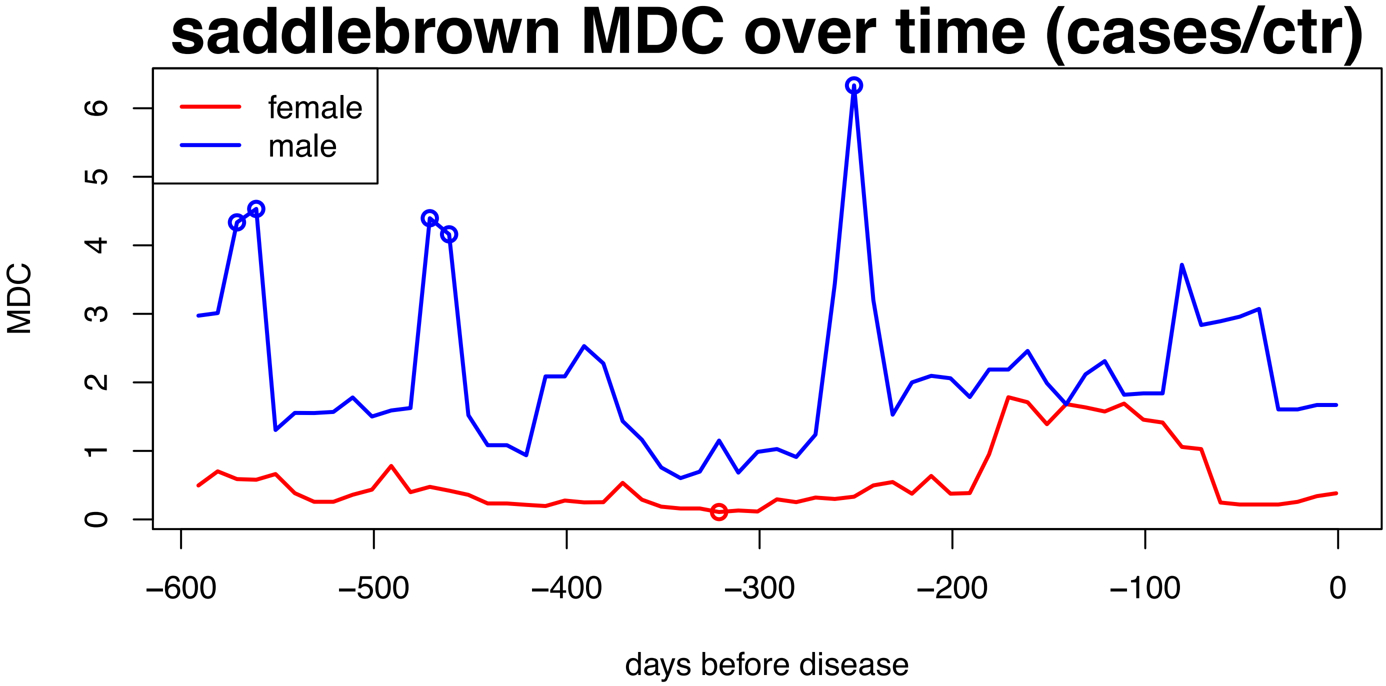

saddlebrown MDC over time (cases/ctr)
female
male
MDC
0
1
2
3
4
5
6
−600
−500
−400
−300
−200
−100
0
days before disease

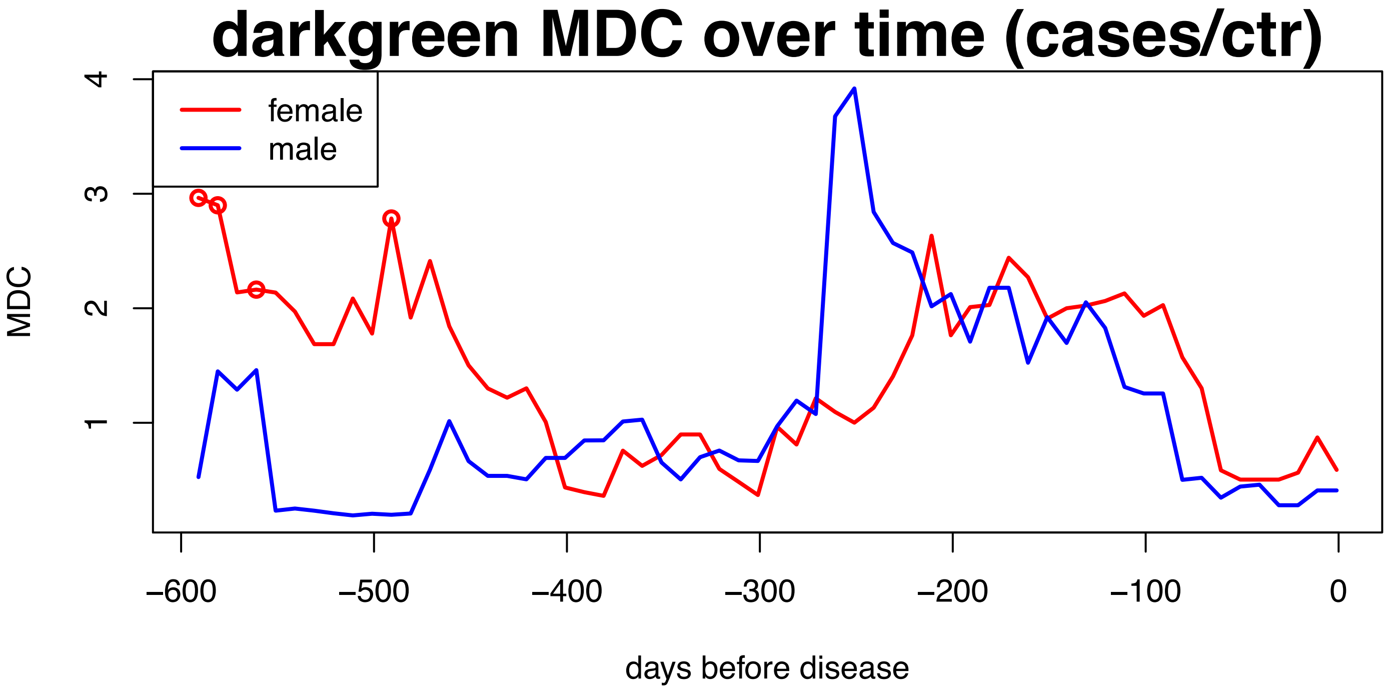
darkgreen MDC over time (cases/ctr)
female
male
MDC
4
3
2
1
−600
−500
−400
−300
−200
−100
0
days before disease

lightcyan MDC over time (cases/ctr)

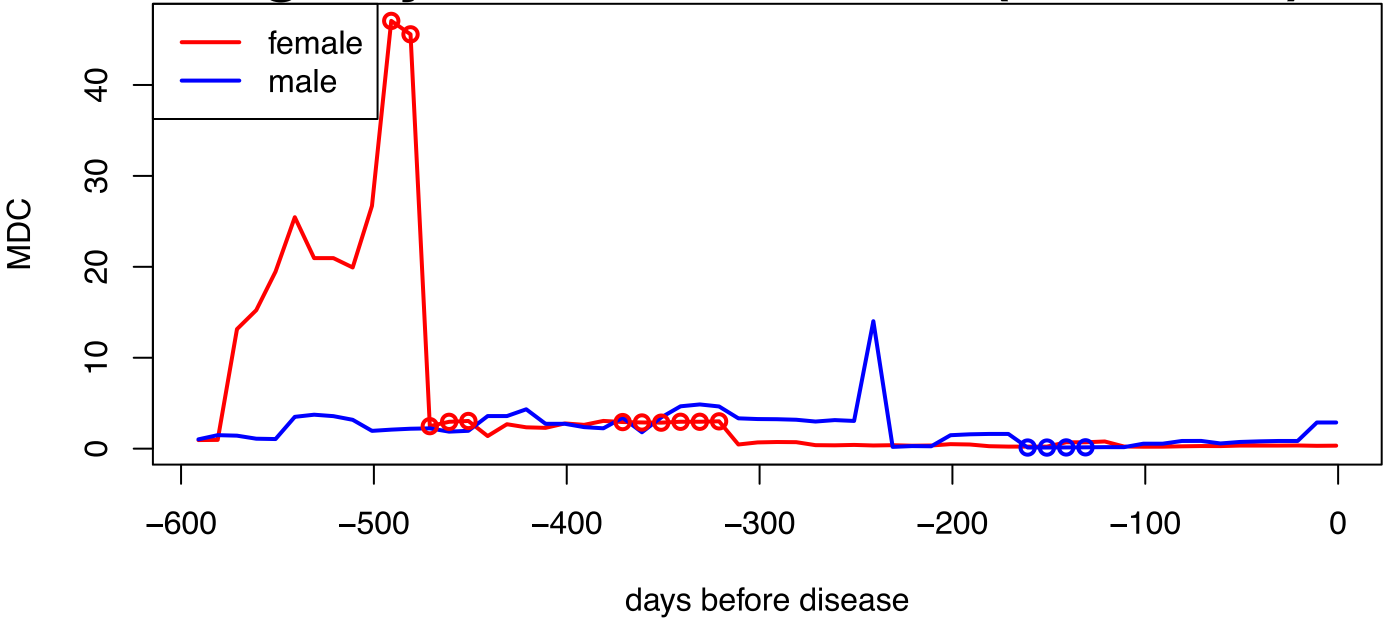

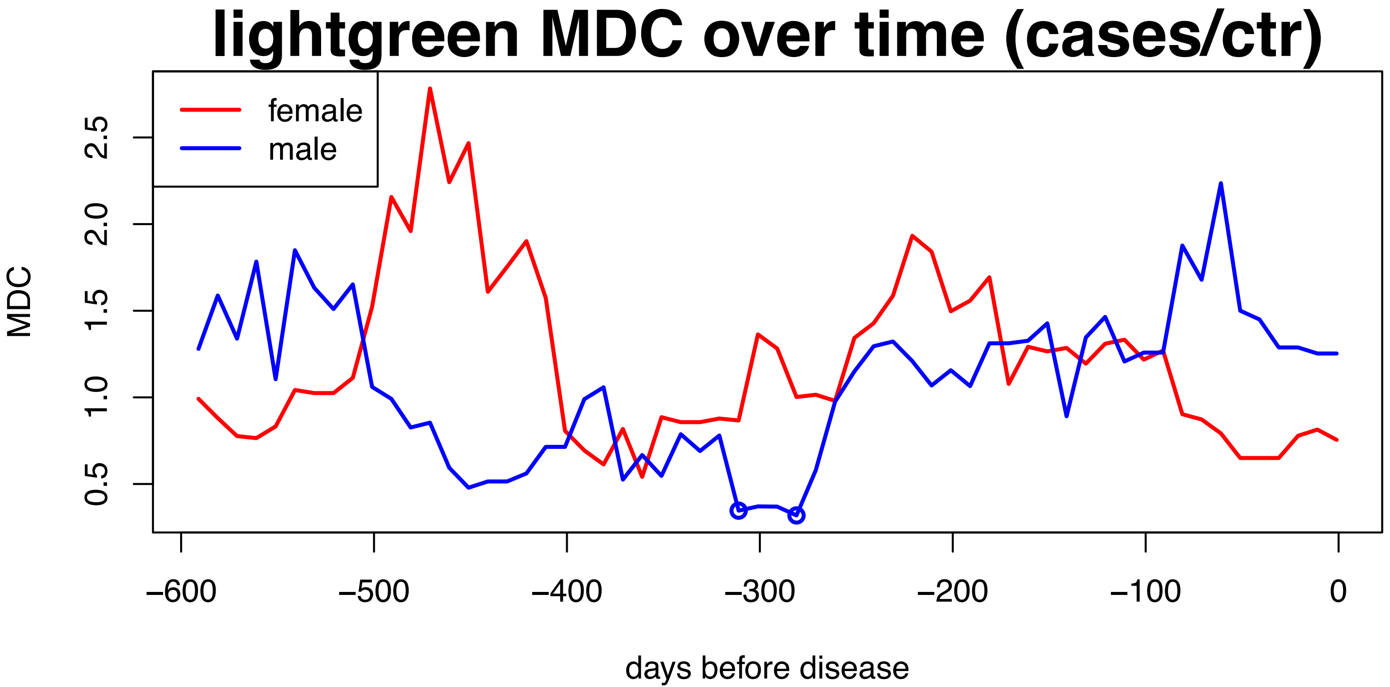
lightgreen MDC over time (cases/ctr)
female
male
MDC
0.5
1.0
1.5
2.0
2.5
−600
−500
−400
−300
−200
−100
0
days before disease

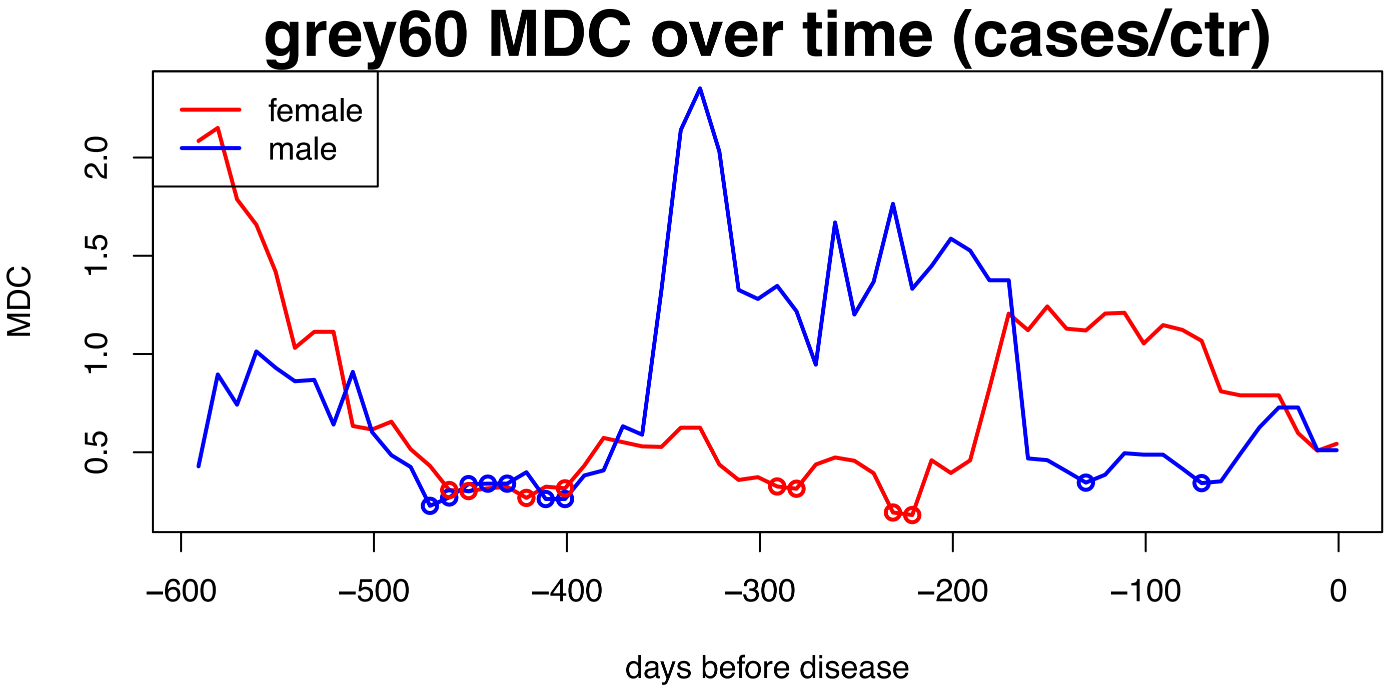

grey60 MDC over time (cases/ctr)
female
male
MDC
0.5
1.0
1.5
2.0
−600
−500
−400
−300
−200
−100
0
days before disease

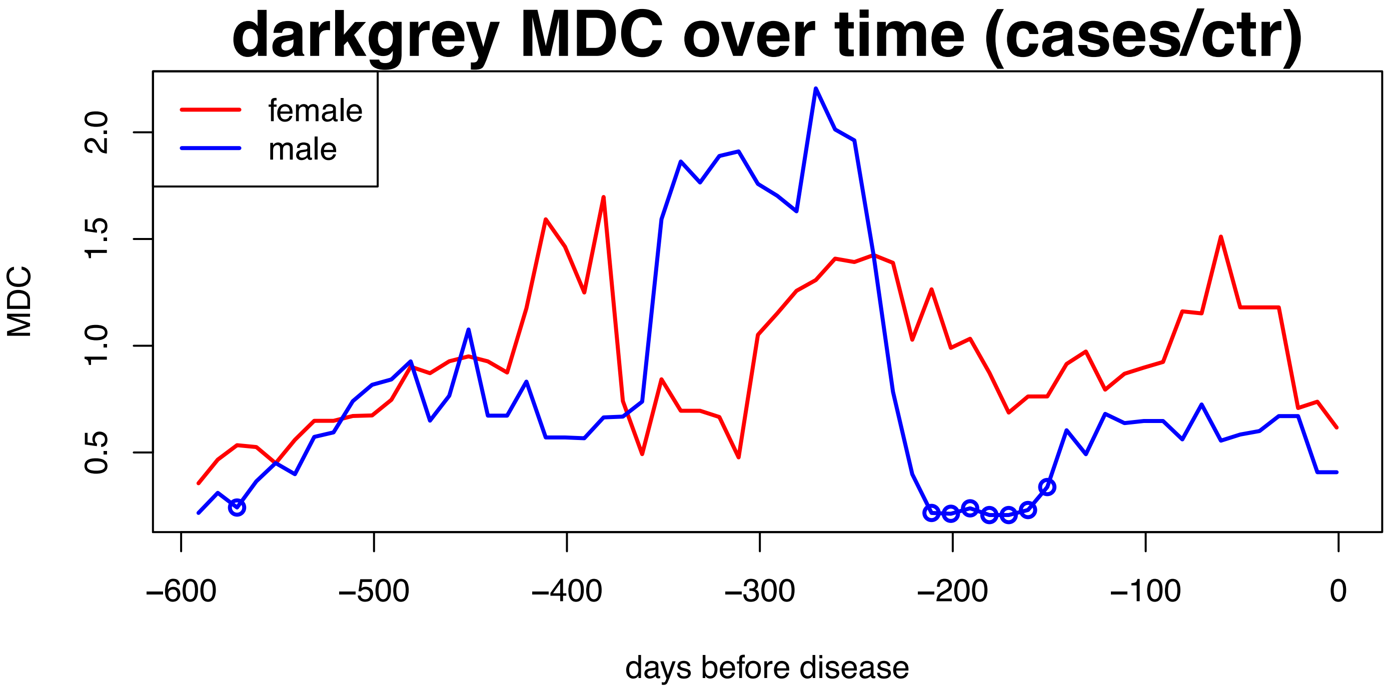
darkgrey MDC over time (cases/ctr)
female
male
MDC
0.5
1.0
1.5
2.0
−600
−500
−400
−300
−200
−100
0
days before disease

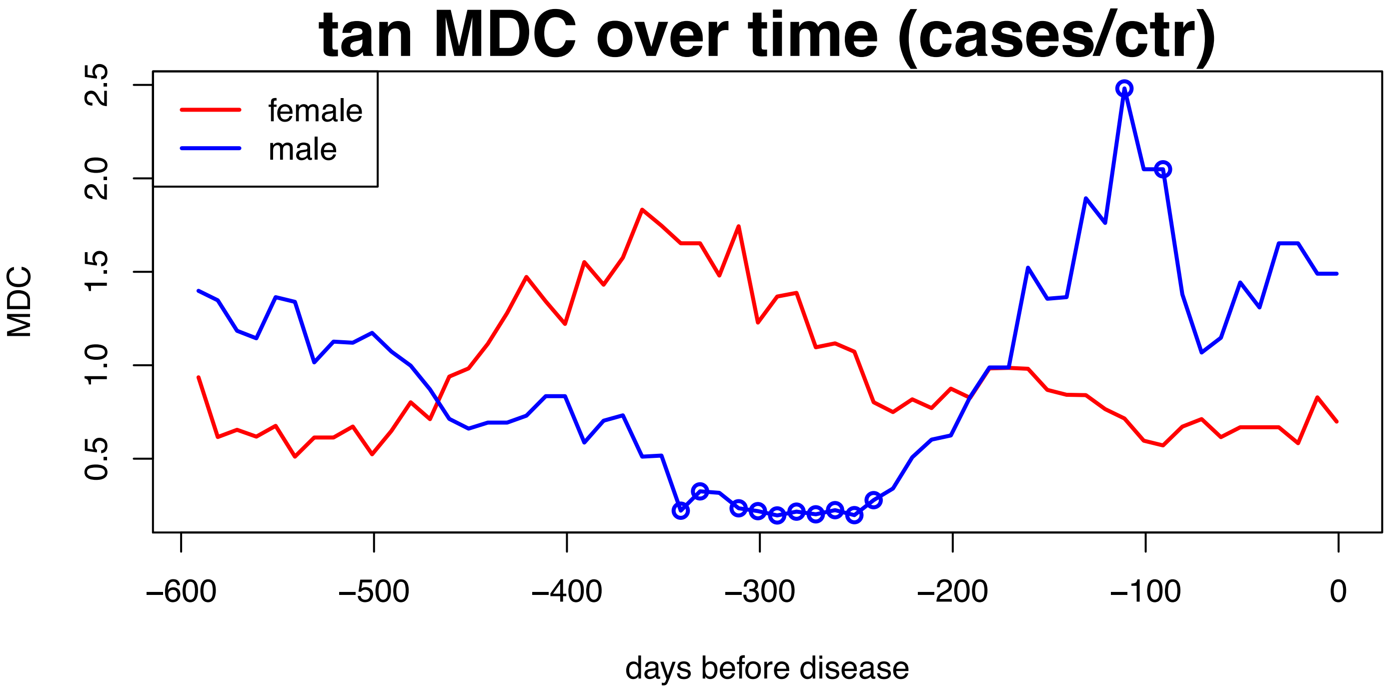
tan MDC over time (cases/ctr)
female
male
MDC
0.5
1.0
1.5
2.0
2.5
−600
−500
−400
−300
−200
−100
0
days before disease

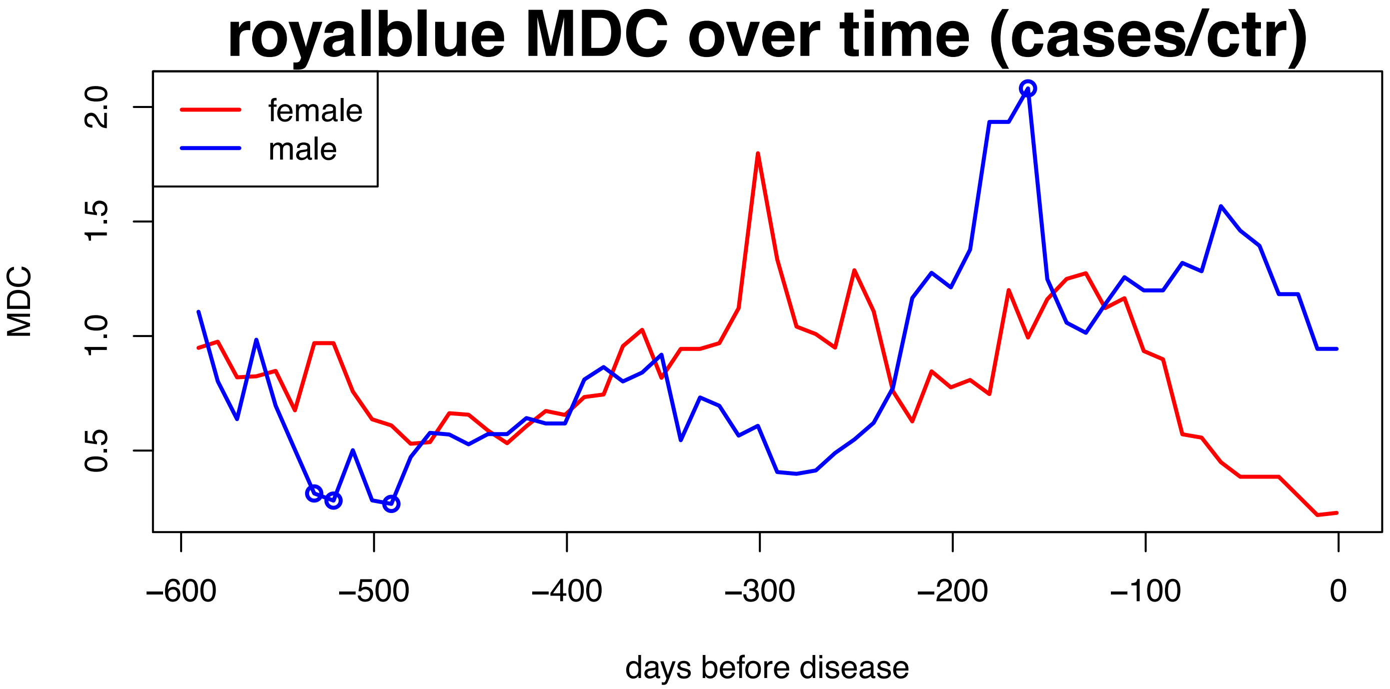

royalblue MDC over time (cases/ctr)
female
male
MDC
0.5
1.0
1.5
2.0
−600
−500
−400
−300
−200
−100
0
days before disease

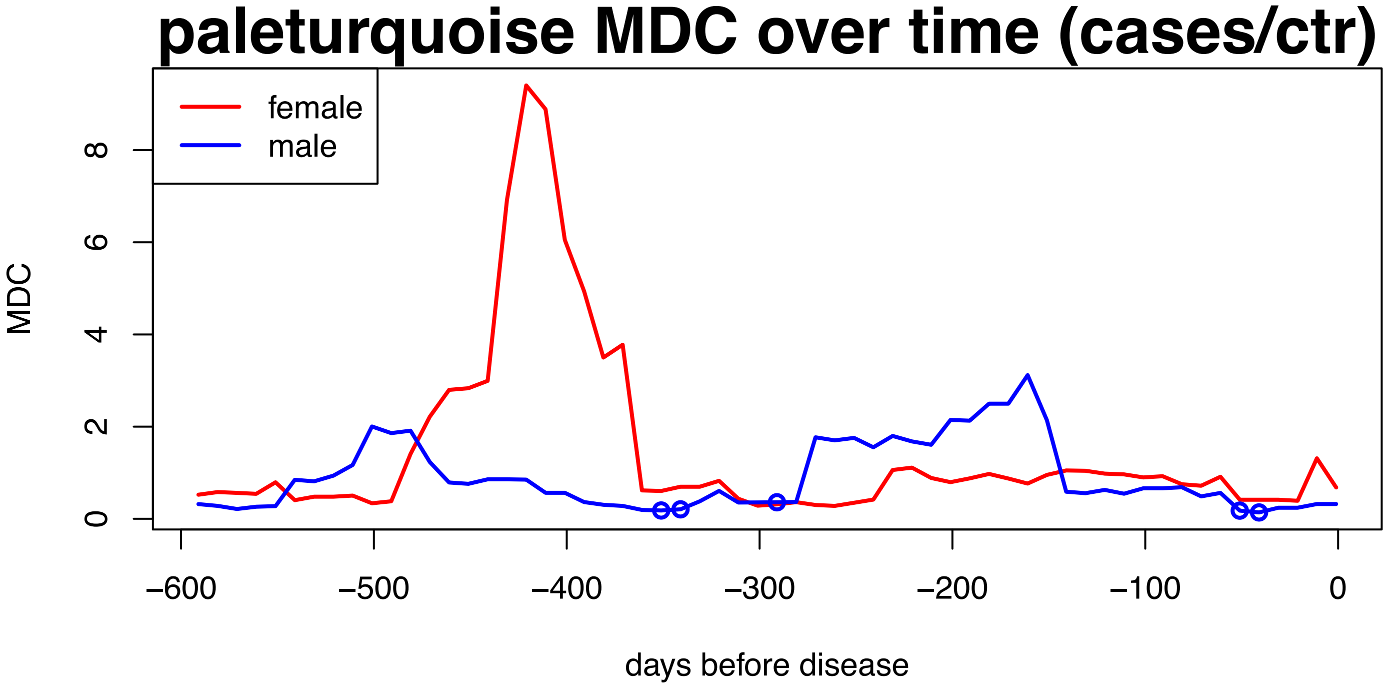
paleturquoise MDC over time (cases/ctr)
female
male
MDC
0
2
4
6
8
−600
−500
−400
−300
−200
−100
0
days before disease

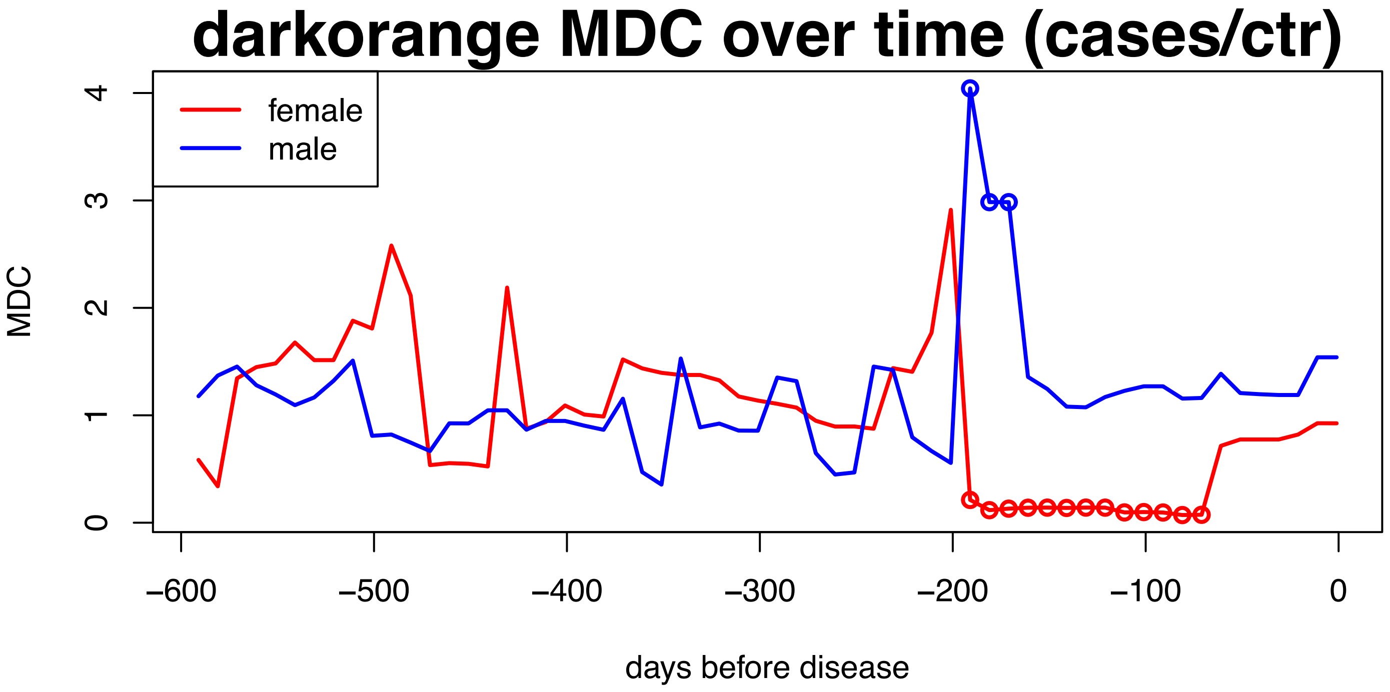

darkorange MDC over time (cases/ctr)
female
male
MDC
0
1
2
3
4
−600
−500
−400
−300
−200
−100
0
days before disease

yellowgreen MDC over time (cases/ctr)

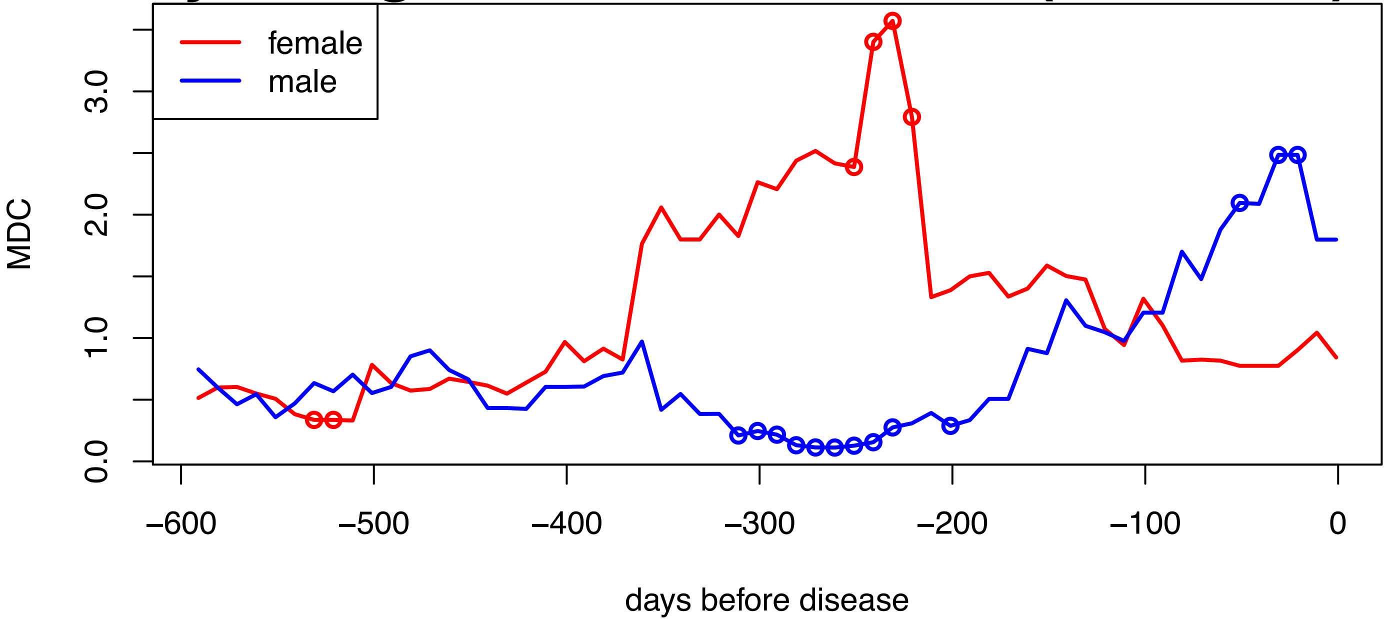

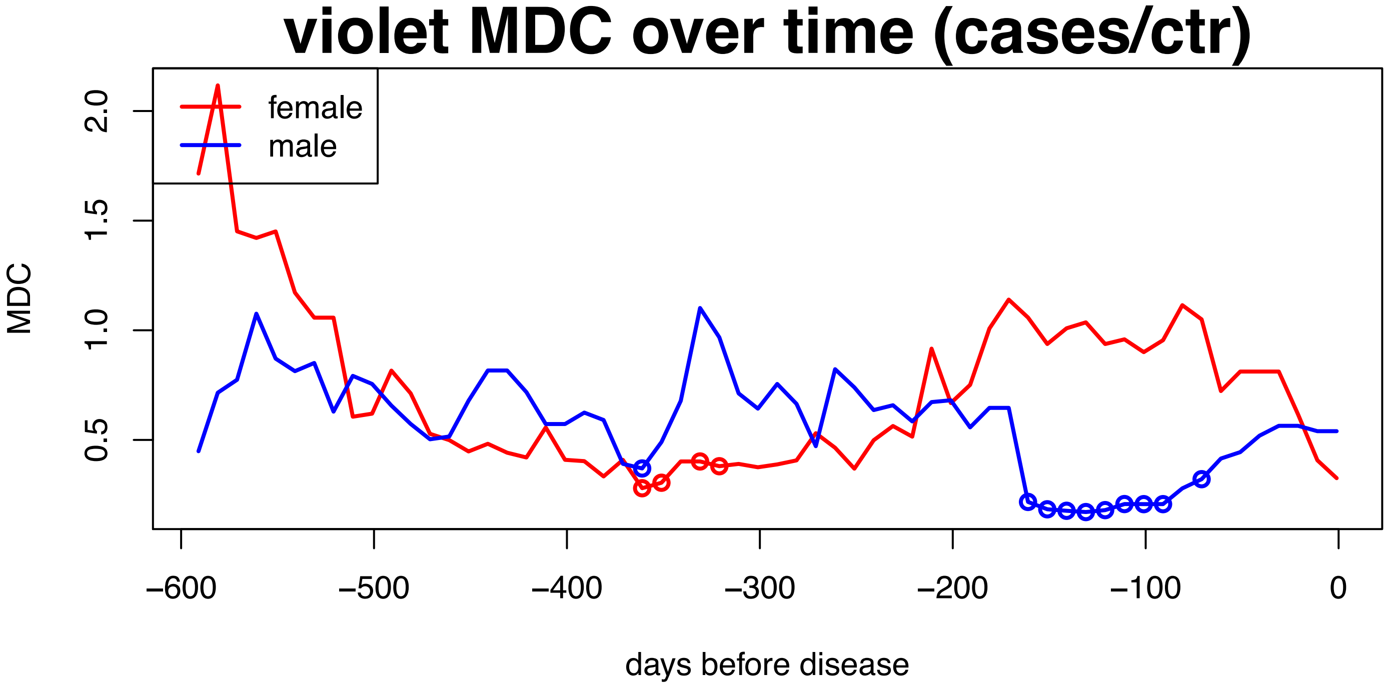

violet MDC over time (cases/ctr)
female
male
MDC
2.0
1.5
1.0
0.5
−600
−500
−400
−300
−200
−100
0
days before disease

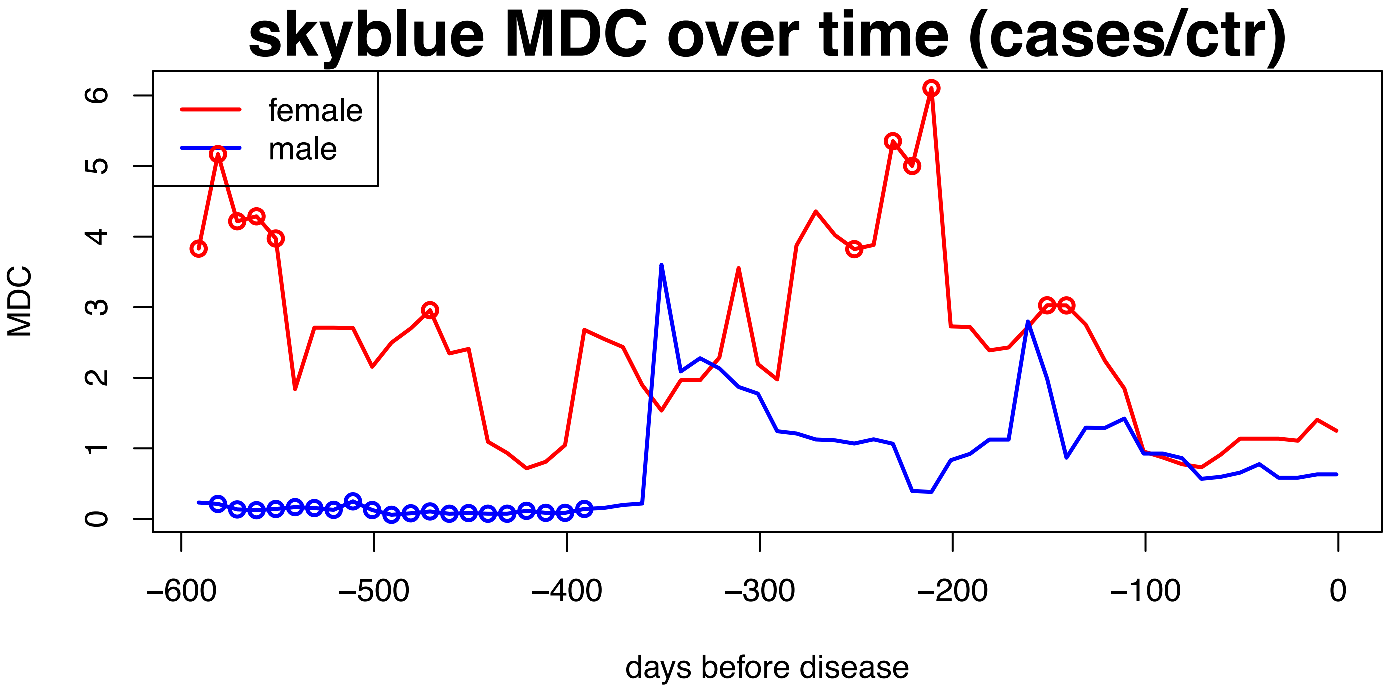

skyblue MDC over time (cases/ctr)
female
male
MDC
0
1
2
3
4
5
6
−600
−500
−400
−300
−200
−100
0
days before disease

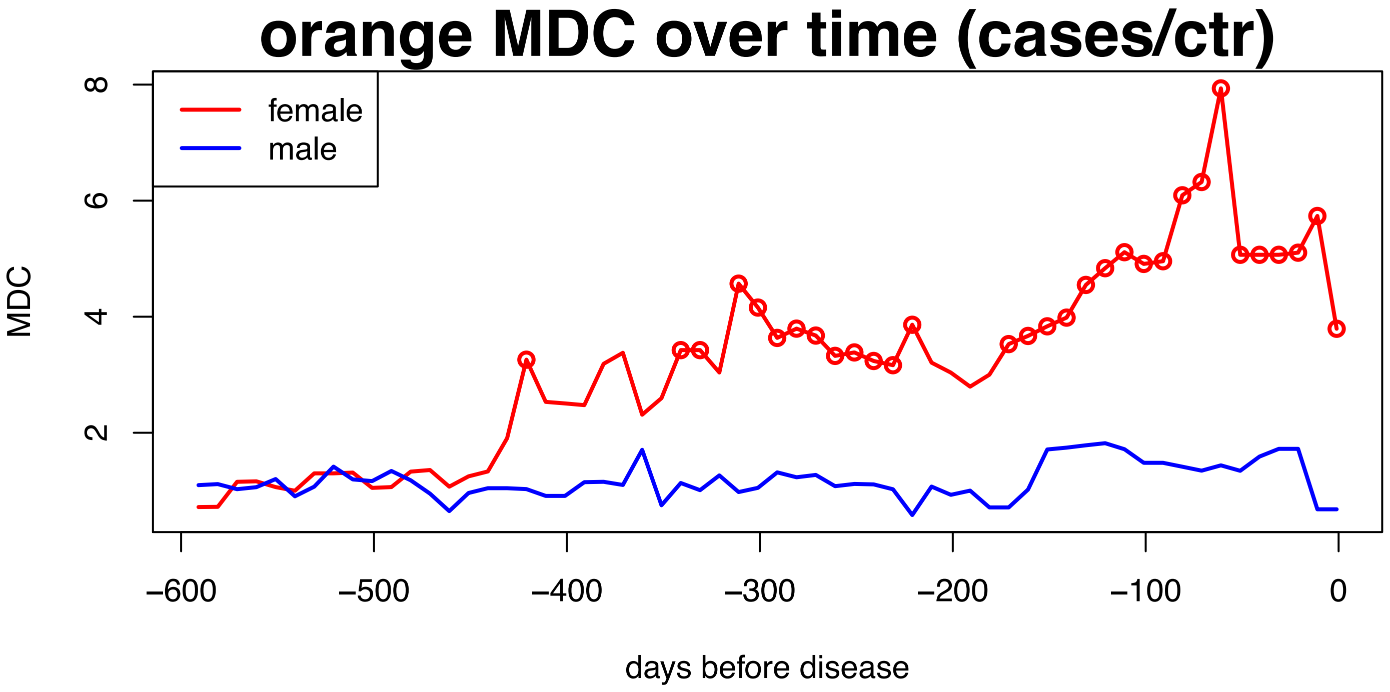
orange MDC over time (cases/ctr)
female
male
MDC
2
4
6
8
−600
−500
−400
−300
−200
−100
0
days before disease

darkmagenta MDC over time (cases/ctr)

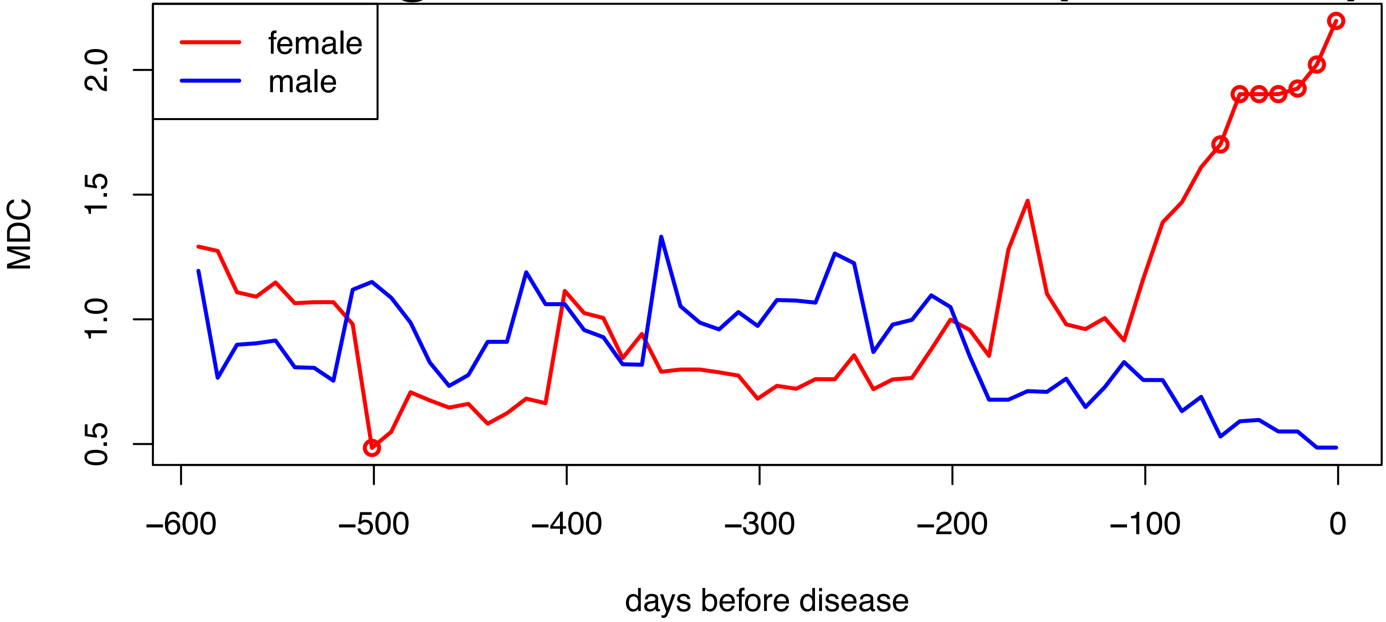

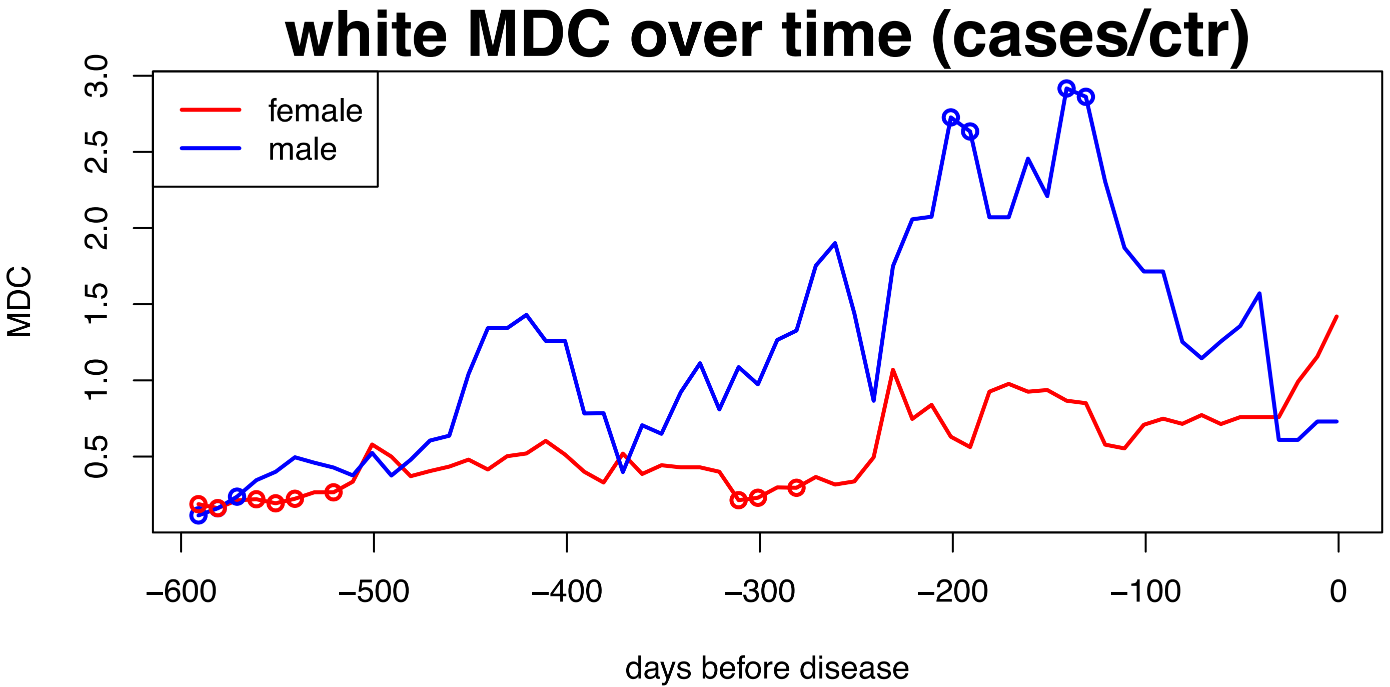
white MDC over time (cases/ctr)
female
male
MDC
0.5
1.0
1.5
2.0
2.5
3.0
−600
−500
−400
−300
−200
−100
0
days before disease

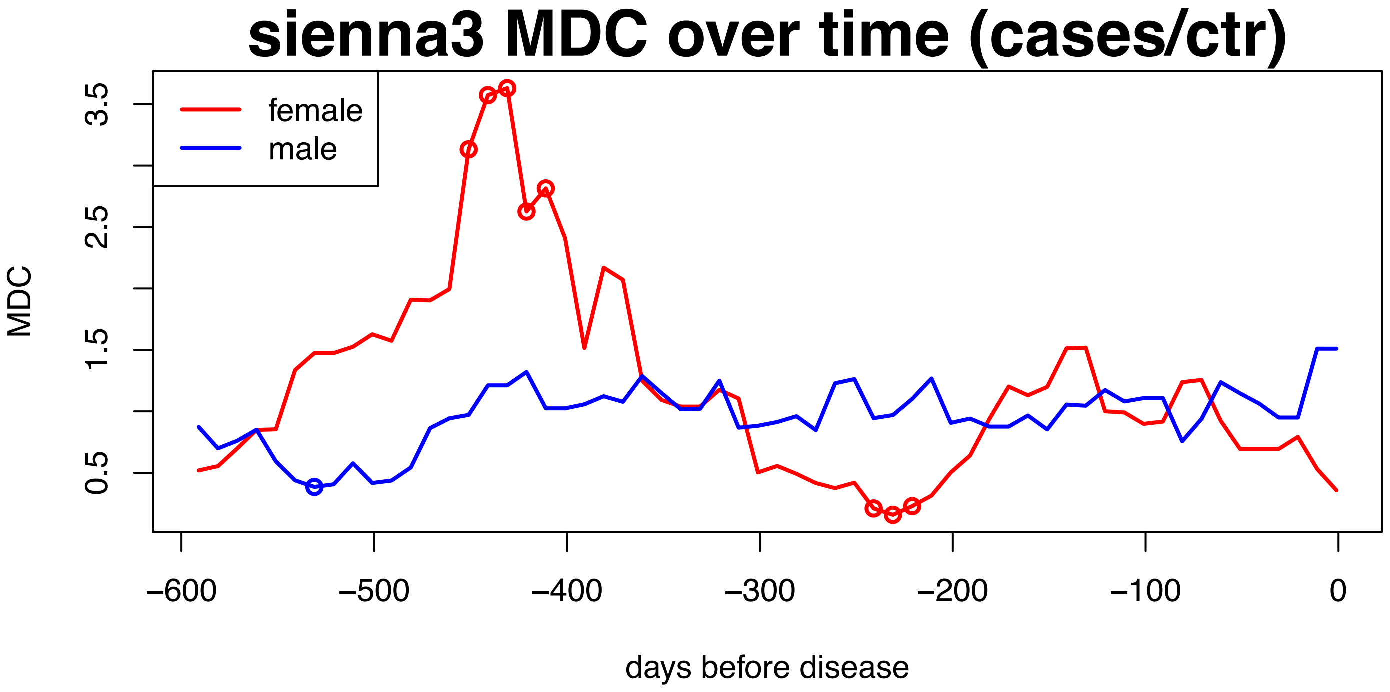
sienna3 MDC over time (cases/ctr)
female
male
MDC
0.5
1.5
2.5
3.5
−600
−500
−400
−300
−200
−100
0
days before disease

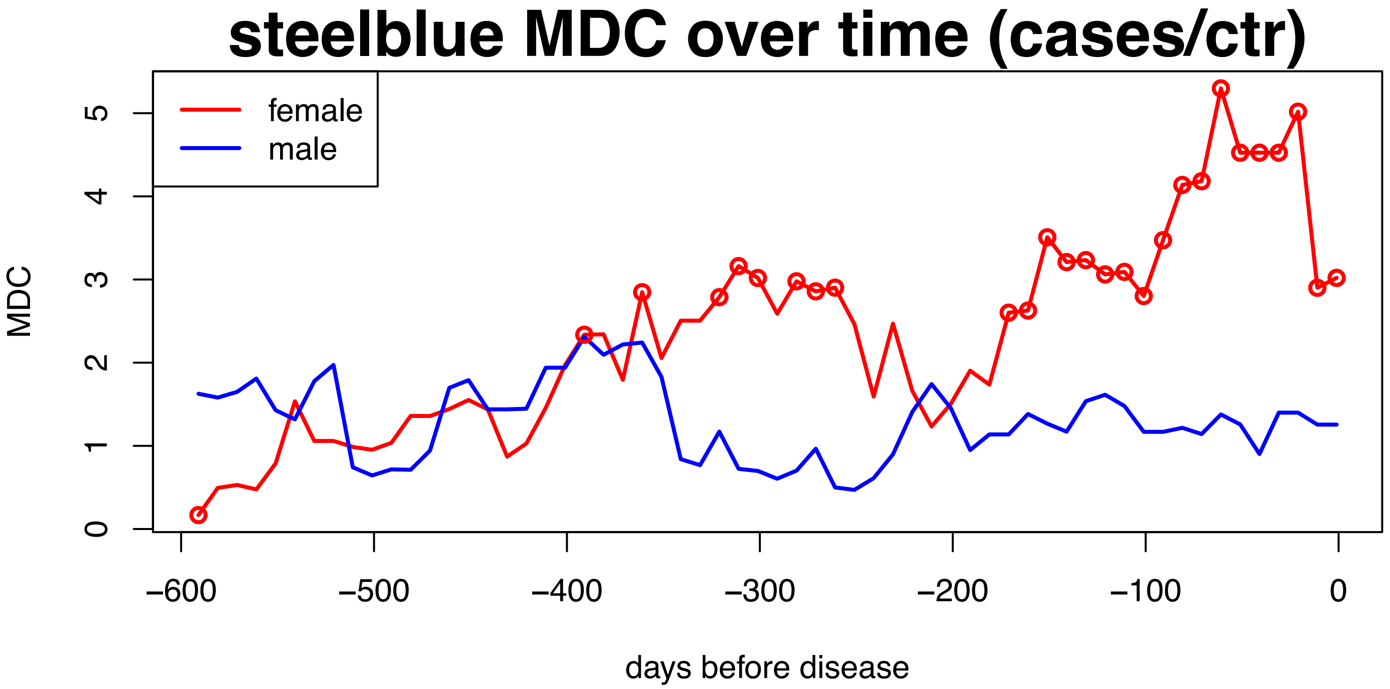
steelblue MDC over time (cases/ctr)
female
male
MDC
0
1
2
3
4
5
−600
−500
−400
−300
−200
−100
0
days before disease

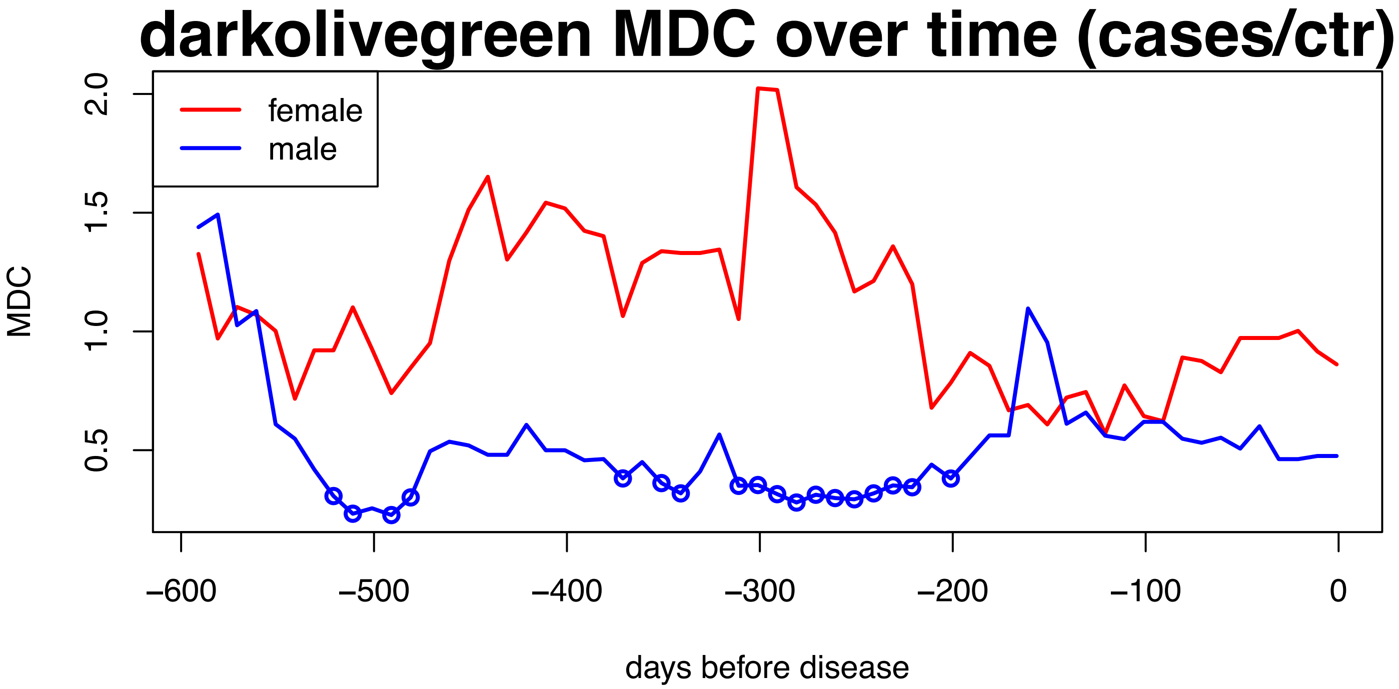

darkolivegreen MDC over time (cases/ctr)
female
male
MDC
2.0
1.5
1.0
0.5
−600
−500
−400
−300
−200
−100
0
days before disease
