# Supplementary material for: Dynamic changes in immune gene co-expression networks predict development of type 1 diabetes
Source: Sci Rep. 2021 Nov 22;11:22651. doi: 10.1038/s41598-021-01840-z (PMC8609030; doi:10.1038/s41598-021-01840-z)
Supplement: Supplementary file 2 — Supplementary Figure 2. [file 41598_2021_1840_MOESM2_ESM.pdf]

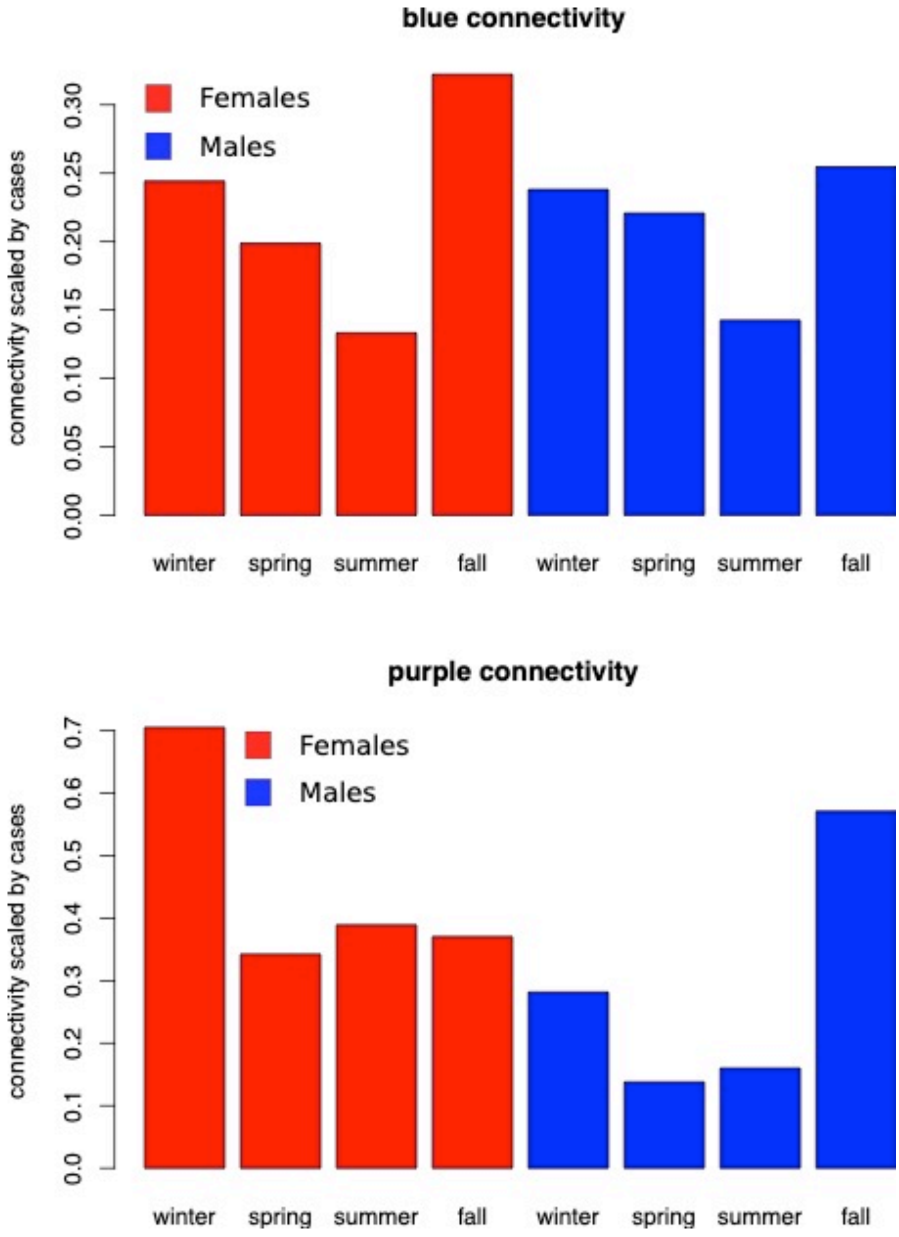

Supplementary Figure 2: Module connectivity by season. Connectivity in controls is scaled by connectivity in cases. Connectivity in females is shown in red, connectivity in males is shown in blue. Winter is defined as the months December, January and February, Spring: March, April and May, Summer: June, July, August, and Fall: September, October and November.
