# Supplementary material for: Dynamic changes in immune gene co-expression networks predict development of type 1 diabetes
Source: Sci Rep. 2021 Nov 22;11:22651. doi: 10.1038/s41598-021-01840-z (PMC8609030; doi:10.1038/s41598-021-01840-z)
Supplement: Supplementary file 3 — Supplementary Figure 3. [file 41598_2021_1840_MOESM3_ESM.pdf]

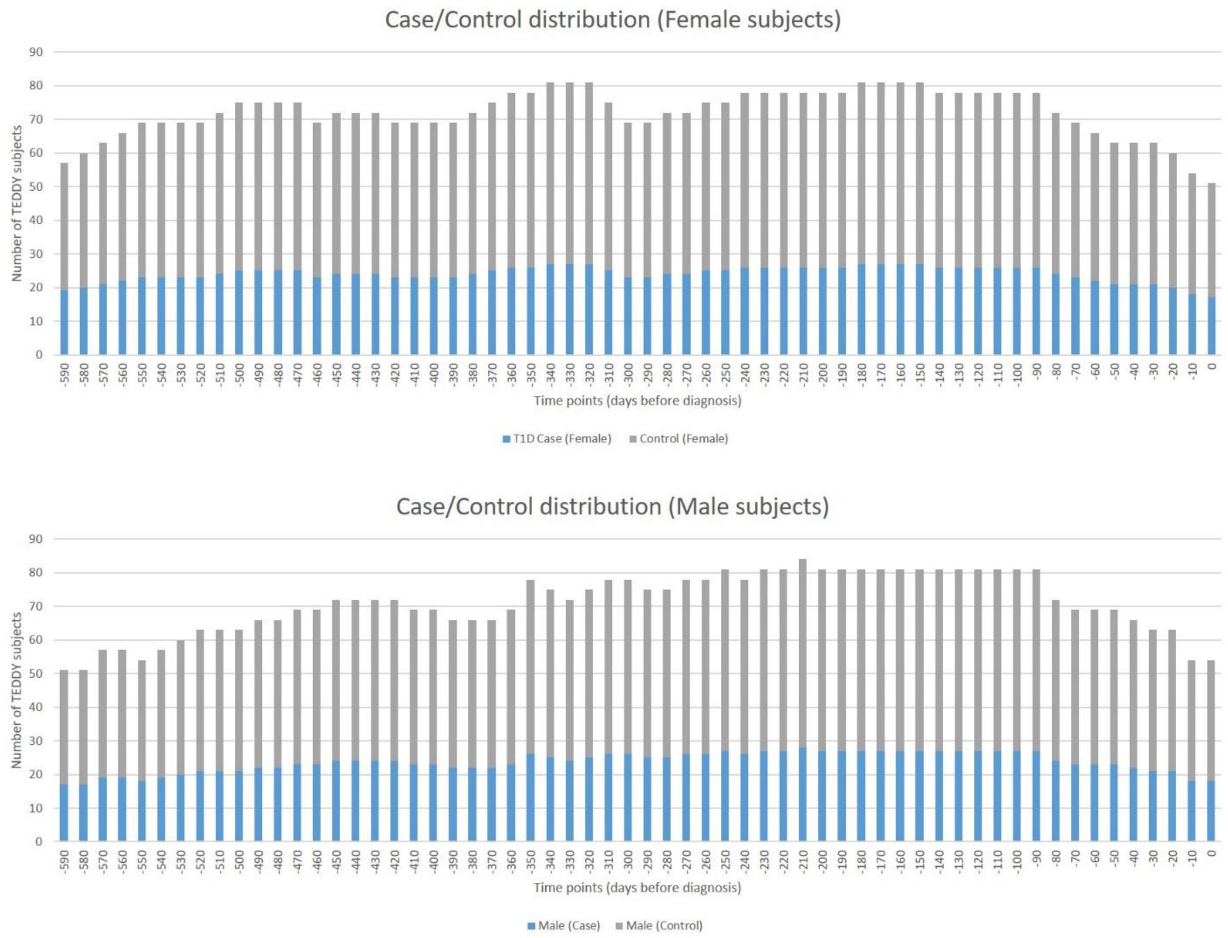

Supplementary Figure 3: T1D case control distribution at each time-point. T1D cases were grouped based on their offset in days to T1D diagnosis. In steps of 10 days, gene expression data (sample) collected from each individual closest to a particular time point was identified. For each T1D sample, at each time point, two control samples were identified that was matched on age and gender. The top panel shows sample distribution at each time point for samples collected from female study subjects and the lower panel shows sample distribution for male study subjects. In both panels, T1D case samples are marked with blue and control samples are marked with grey.
